# Enhanced chimp optimization algorithm for high level synthesis of digital filters

**DOI:** 10.1038/s41598-022-24343-x

**Published:** 2022-12-10

**Authors:** Mandeep Kaur, Ranjit Kaur, Narinder Singh

**Affiliations:** 1 Department of Electronics and Communication Engineering, Punjabi University, Patiala, India; 2 Department of Mathematics, Punjabi University, Patiala, India

**Keywords:** Engineering, Mathematics and computing

## Abstract

The HLS of digital filters is a complex optimization task in electronic design automation that increases the level of abstraction for designing and scheming digital circuits. The complexity of this issue attracting the interest of the researcher and solution of this issue is a big challenge for the researcher. The scientists are trying to present the various most powerful methods for this issue, but keep in mind these methods could be trapped in the complex space of this problem due to own weaknesses. Due to shortcomings of these methods, we are trying to design a new framework with the mixture of the phases of the powerful approaches for high level synthesis of digital filters in this work. This modification has been done by merging the chimp optimizer with sine cosine functions. The sine cosine phases helped in enhancing the exploitation phase of the chimp optimizer and also ignored the local optima in the search area during the searching of new shortest paths. The algorithms have been applied on 23-standard test suites and 14-digital filters for verifying the performance of the algorithms. Experimental results of single and multi-objective functions have been compared in terms of best score, best maxima, average, standard deviation, execution time, occupied area and speed respectively. Furthermore, by analyzing the effectiveness of the proposed algorithm with the recent algorithms for the HLS digital filters design, this can be concluded that the proposed method dominates the other two methods in HLS digital filters design. Another prominent feature of the proposed system in addition to the stated enhancement, is its rapid runtime, lowest delay, occupied area and lowest power in achieving an appropriate response. This could greatly reduce the cost of systems with broad dimensions while increasing the design speed.

## Introduction

High-level synthesis (HLS) is a hot topic and a design process in which a high-level, functional description of a design is automatically compiled into an register transfer level setup that meets some user-defined design constraints. In re-timing, any difficult optimization problem can be alienated into independent sub-problems. If any problem or function have divided into ($m-1$) flip-flops, in that decreasing the overall time by these factor (*m*). Generally this process is apply to obtain synchronous circuits (SYC). And re-timing method is used to enhanced the speed of the SYC without changing the latency and functionality^1^. A re-timing method can also be generalized to locate non-critical gates that can be operated with low supply voltages to reduce overall system power consumption. The various re-timing strategies have developed by the researchers in the literature for the issues of power consumption such as^2–4^ respectively. In Ref.^5^, have developed a new framework for retiming and for the digital filters the digital signal processing blocks based evolutionary computation process. During this strategy takes the inputs form the user in the form of data flow graphs or matrices or obtains all optimal solutions in the search domain.

In addition, the high level of synthesis of digital filtering is a big challenge for the engineers. In this issue researchers have solved various complacence’s of the power consumption in the terms of maximized frequency and reducing area. Last few decades, various recent MA’s are applied for tackling of these kinds of issues. And for the demand of the future more robust algorithms are developing, so that these can be used to fulfil the future requirements. For highlighting the important of this work we are discussing more by the following some references.

Digital filter can be categorized into two main groups: Finite Impulse Response (FIR)^6^ and Infinite Impulse Response (IIR)^7^. Filters^8^, are the major crucial systems in most electronic and computing machines. Filtering aims to extract information about the most relevant interesting signals, either by extracting frequency components or by separating desired components from unwanted signals or eliminating noise. As per view of the mathematical, the designing of the digital filters according to a specified criterion, can be formulated as an optimization function where need to find the best suites specifications. Thus, the various robust stochastic algorithms during the last few decades has developed the different kinds of metaheuristic algorithms (MHAs). These are sophisticated MA’s, they are often a better alternative to traditional nature inspired algorithms, giving an excellent trade off amid the computing time and optimal solution’s quality, particularly for complex optimization functions or large dimension issues. Generally, the metaheuristic algorithm can be divided into different phases as per sources of inspiration^9^: (1) natural phenomena (NP) methods that imitate the principles of physics and chemistry. (2) Evolutionary methods that follow the natural evolution processes found in nature; and (3) swarm intelligence (SI) methods, including population-based algorithms that mimic the social behavior of insects or animals. In particular, Chimp Optimizer^10^, is a new population based method that has already started attracting attention. In this study we are trying to present the novel hybrid method with the mixture of two powerful algorithms such as ChOA with Sine Cosine, it is called SChoA. These powerful features of two methods play an important role for trapping the best solutions in the global search space. The sine cosine features helped in enhancing the exploitation phase of the chimp optimizer and also ignored the local optima in the search area during the searching of new shortest paths. The main objective of this study is to introduce the powerful method for considering the high level synthesis of the complex dimension digital filters. The experimental numerical and statistical outcomes show that the proposed method performance is superior to other well-known MHAs in the literature. In summary, the main contributions of this paper are:

- A novel improved algorithm called SChoA method that includes features from Chimp and sine cosine functions is proposed.
- The proposed method performance is developed for high-level synthesis (HLS) of datapaths in digital filters.
- proposed strategy is developed for solving single and multi-objective issues.
- Statistical and qualitative numerical result analyses assess the performance of the proposed method compared to other competitive algorithms.

The remainder of this paper is as follows: Th related works have been described in “Related work”. An overview about the Chimp Optimizer Algorithm (ChoA) are presented in “Chimp optimizer algorithm (ChoA)” respectively. “Problem formulation”, illustrates the all details of the high level synthesis (HLS) of the digital filters. “Modified SChoA version for high level synthesis of digital filters” describes the mathematical model of the proposed SChoA algorithm. “Results analysis and discussion of 23-standard test suites” and “Simulations and results of digital filters” presents the results and their analyses obtained by the proposed strategy and the competitor algorithms. “Conclusion and future work” concludes the paper.

## Related work

MHAs recently are playing an most important role for digital filters issues. In fact, MHAs have provided extraordinary performances in several practical problems of a broad domain of applications, e.g., feature selection^11–14^, optimization problems^15^, constrained engineering problems^16^, traveling salesman problems^17^, Case study Email spam detection^18^ respectively. For superior efficiency of the MA’s, various robust population based algorithms have been developed in the last few decades. This growing interest in population based algorithms coincides with the need for more efficient algorithms for finding the best solution’s of the complicated optimization functions. Some of these metaheuristic methods, including Genetic algorithms (GAs)^19^, Particle swarm optimization (PSO)^20^, Henry gas solubility optimization (HGSO)^21^, Simulated annealing algorithm (SA)^22^, Archimedes optimization algorithm (AOA)^23^, Cuckoo Search (CS) algorithm^24^, Lévy flight distribution (LFD)^25^ and Chimp optimizer algorithm (ChoA)^10^, One Half Personal Best Position Particle Swarm Optimizations (OHGBPPSO)^26^, Personal Best Position Particle Swarm Optimization (PBPPSO)^27^, Half Mean Particle Swarm Optimization Algorithm (HMPSO)^28^, HAGWO^29^, Hybrid Particle Swarm Optimization (HPSO)^30^, HPSOGWO^31^, Hybrid MGBPSO-GSA^32^, HGWOSCA^33^, MGWO^34^, MVGWO^35^, HSSAPSO^36^, SChoA^37^, HSSASCA^38^, HSSAHHO^39^, Hybrid Chimp-Cuckoo search algorithm (ChCS)^40^, An enhanced chimp optimization algorithm for optimal degree reduction of Said-Ball curves^41^, Dynamic levy flight chimp optimization^42^, A weighted chimp optimization algorithm^43^, Niching chimp optimization for constraint multimodal engineering optimization problems^44^, Fuzzy-ChOA: an improved chimp optimization algorithm for marine mammal classification using artificial neural network^45^, Optimization of constraint engineering problems using robust universal learning chimp optimization^46^, Multi-Objective chimp Optimizer:An innovative algorithm for Multi-Objective problems^47^, An enhanced chimp optimization algorithm for continuous optimization domains^48^ and the SCA method was developed by Mirjalili et al.^49^ for real world optimization issues. This algorithm is the most robust method for complex issues. It has played an important role in the modifications of the basis algorithm for presenting the new one enhanced methods. The SCA method has been designed by sine and cosine trigonometric functions. These functions play an important role for superior exploration and exploitation phases of the algorithm. The following mathematical formulations are applied in this method for finding the new one position in the search domain.

2.1 $$\begin{aligned} \vec {x}_{i}^{t+1}= & {} \vec {x}_{i}^{t} +r_{1} \times \sin \left( r_{2} \right) \times \left| r_{3} \times l_{i}^{t} -\vec {x}_{i}^{t} \right| \end{aligned}$$

2.2 $$\begin{aligned} \vec {x}_{i}^{t+1}= & {} \vec {x}_{i}^{t} +r_{1} \times \cos \left( r_{2} \right) \times \left| r_{3} \times l_{i}^{t} -\vec {x}_{i}^{t} \right| \end{aligned}$$

where $$\vec {x}_{i}^{t} $$, $$r_{1} ,r_{2} ,r_{3} \in [0,1]$$ are illustrates the current position and random numbers and $$l_{i} $$ is targeted global optimal result. The above mathematical Eqs. (2.1)–(2.2) uses $$0.5\le r_{4} <0.5$$ setting for exploitation and exploration.

2.3 $$\begin{aligned} \vec {x}_{i}^{t+1} =\left\{ \begin{array}{cc} {\vec {x}_{i}^{t} +r_{_{1} } \times \sin \left( r_{_{2} } \right) \times \left| r_{_{3} } \times l_{i}^{t} -\vec {x}_{i}^{t} \right| } &{} {,\, \, \, r_{_{4} } <0.5} \\ {\vec {x}_{i}^{t} +r_{_{1} } \times \cos \left( r_{_{2} } \right) \times \left| r_{3} \times l_{i}^{t} -\vec {x}_{i}^{t} \right| } &{} {\, ,\, \, r_{_{4} } \ge 0.5} \end{array}\right. \end{aligned}$$

Recently, digital filtering is a big challenging optimization function. It is worth mentioning, that with the help of the robust nature inspired algorithms the various drawbacks of the digital filters have been resolved such as processing time and enhances the characteristics of the designed digital filters etc^50,51^. Mohanty et al.^52^, have developed a distributed arithmetic approach for reconfigurable block-based FIR filter, which is scalable for larger block-sizes and higher filter-lengths. A new algorithm based on African vultures’ lifestyle is developed by Abdollahzadeh et al.^53^. This strategy simulates African vultures’ foraging and navigation behaviors. The performance of this approach is verified through 36 standard test suites. Abdollahzadeh et al.^54^ has presented the algorithm by gorilla troops’ social intelligence in nature, it is known as Gorilla Troops Optimizer (GTO). In this work, the gorillas’ collective life is scientifically framed, and new mechanisms are intended to execute exploitation and exploration. The robustness of the presented approach has been tested through 52 standard suites and engineering functions. A new approach that is stimulated through farmland fertility in nature is introduced by Shayanfar and Gharehchopogh^55^, which has been assessed by utilizing the complex issues. In addition, the overview of Whale Optimization Algorithm, Spotted Hyena Optimizer, symbiotic organisms search algorithms and its applications is presented by Gharehchopogh et al.^56^. Also, Luis and Arribas^57^, have proposed a new approach for the design of digital frequency selective FIR filters using an flowers pollination algorithm (FPA), with a novel multiple fitness function, to get optimised filter coefficients that best approximate ideal specifications. Yadav et al.^58^, applied grasshopper optimization algorithm (GOA) to design a linear phase finite impulse response (FIR) low pass, high pass, band pass, and band stop filters. proposed methodology target to reach minimum absolute error difference fitness function, through selecting optimal filter coefficients. In^59^, the effectiveness of employing the swarm intelligence (SI) based and population-based evolutionary computing techniques is investigated for determining the optimal solutions to the FIR filter design problem.

The research of applying nature inspired algorithms to digital filters design has attracted much attention in last few years due to its utilization in a wide range of complex optimization functions^60^. In general, digital filter is an optimization issue, in last few decades, regarding this issue various new strategies have been developed by the researchers for instance, such as; Lagos-Eulogio, Pedro, et al.^61^, have developed a new hybrid algorithm based on the combination of cellular particle swarm optimization (PSO) and differential evolution (DE) called CPSO-DE for the optimal parameter estimation of IIR digital filters. Wanga et al.^62^ have presented a novel design method that used a membrane computing method to design an optimal digital filter, their strategy that employed a tissue-like membrane system with ring-shaped topology structure. Panda et al.^63^, have presented an IIR system identification using the cat swarm optimization (CSO). Wang et al.^64^ have developed a framework called two-stage ensemble memetic algorithm (TSMA), TSMA employed to synthesize the strengths of the evolutionary global search and local search techniques. The proposed TSMA applied to design high-order digital IIR filters, experimental results compared to 6 state-of-the-art algorithms. Kaur et al.^65^, have applied a new model to optimize the magnitude response and the phase response based on the greedy search method, binary successive approximation (BSA) and evolutionary search (ES) simultaneously, along with finding the lowest order of the filter. Kumar and Rawat^66^ have employed cuckoo search algorithm (CSA), to get optimal coefficients of a fractional delay IIR (FD-IIR) filter, and to have an ideal frequency response characteristics. Upadhyay, P., et al.^67^, have combined the Differential Evolution (DE) with Wavelet Mutation (DEWM) to IIR system identification problem. Also, to develop proper IIR filter designing method as a multi-objective optimization problem, Wang, Yu, Bin Li, and Yunbi Chen^68^, have proposed a new local search operator enhanced multi-objective evolutionary algorithm (LS-MOEA). Saha et al.^69^, have Presented a hybrid method of Gravitational Search Algorithm (GSA) and Wavelet Mutation (WM) called GSAWM, which applied on design of an 8th-order IIR filter, GSAWM target to achieve better cut-off frequency sharpness, smaller pass band and stop band ripples. Additionally, in^70^, 2-dimensional IIR Filter design method based on hybrid PSO and SA is presented.

## Chimp optimizer algorithm (ChoA)

A novel population based method, is known as chimp optimizer recently originated by Khishe et al.^10^. The method is inspired by sexual motivation and individual intelligence of chimps. And it’s most famous for their group hunting. The hunting approach of this method is differ from the others. In this,strategy has used the four different phases such as driver, chaser, barrier and attacker respectively for searching the best score in the search domain.

All the working steps have been illustrated through the following mathematical formulations;

For chasing and driving the prey, has been used the following Eqs. (3.1)–(3.2);

3.1 $$\begin{aligned}{} & {} D=\left| c.a_{prey}\left( n \right) -ma_{chimp}\left( n \right) \right| \end{aligned}$$

3.2 $$\begin{aligned}{} & {} a_{chimp}\left( n+1 \right) =a_{prey}-a.d \end{aligned}$$

where *n* illustrates the number of generations ,*c*,*m* and *a* are the coefficient vectors. These vectors *c*,*m* and *a* are evaluated through Eqs. (3.3)–(3.4)

3.3 $$\begin{aligned} a= & {} 2.l.r_1-l \end{aligned}$$

3.4 $$\begin{aligned} c= & {} 2.r_2 \end{aligned}$$

3.5 $$\begin{aligned} m= & {} chotic_{value} \end{aligned}$$

where $r_1$ and $r_2$ are illustrated random values lying between $\left[ 0,1 \right]$, *m* is denoted the chotic vector and *l* has use for reducing non-linearly from 2.5 to 0 through the generation process. In this stage the behavior of the search agents has applied through mathematically. Firstly in the initial stage the each search member position is chosen by the given random values. In the next iteration, the first four best solutions are stored for updating the new position of the search member in the search domain. This procedure has been evaluated through the following mathematical Eqs. (3.6-3.9);

3.6 $$\begin{aligned} d_{a}= & {} \left| c_1a_{a}-m_1.x \right| \end{aligned}$$

3.7 $$\begin{aligned} d_{b}= & {} \left| c_2a_{b}-m_2.x \right| \end{aligned}$$

3.8 $$\begin{aligned} d_{c}= & {} \left| c_3a_{c}-m_3.x \right| \end{aligned}$$

3.9 $$\begin{aligned} d_{d}= & {} \left| c_4a_{d}-m_4.x \right| \end{aligned}$$

When the random values are lies amid $\left[ -1,1 \right]$, then the next position of the search member can be in any position amid its current location and the location of the target or prey.

3.10 $$\begin{aligned} x_1= & {} a_{a}-a_1.d_{a} \end{aligned}$$

3.11 $$\begin{aligned} x_2= & {} a_{b}-a_2.d_{b} \end{aligned}$$

3.12 $$\begin{aligned} x_3= & {} a_{c}-a_3.d_{c} \end{aligned}$$

3.13 $$\begin{aligned} x_4= & {} a_{d}-a_4.d_{d} \end{aligned}$$

As per all above mathematical formulations, the position of the search is evaluated by the Eq. (3.14);

3.14 $$\begin{aligned} x_{n+1}=\frac{x_1+x_2+x_3+x_4}{4} \end{aligned}$$

At end, for position updating of each member has used following Eq. 3.15.

3.15 $$\begin{aligned} a_{chimp}\left( n+1 \right) =\left\{ \begin{matrix} a_{prey}\left( n \right) -x.d,&{} if &{} \phi <0.5 \\ chaotic_{value}&{} if &{} \phi >0.5 \end{matrix}\right. \end{aligned}$$

### Pseudocode of chimp optimizer (ChoA)

The pseudocode of ChoA is illustrated through Algorithm 1.
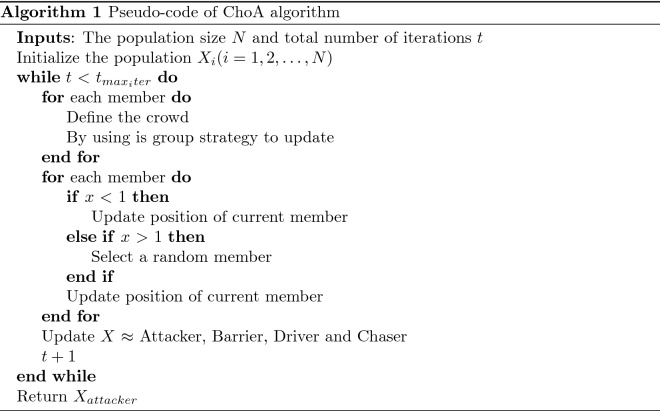


## Problem formulation

Retiming is a robust method for optimization which improves the sequential circuit performance. Generally, this method is applied for changing the positions of delay variables in a circuit without affecting the initial input and end outputs of the circuit. The brief details related of retiming are illustrates through the following subsections;

### Clock period

The main purpose of Retiming is transforming a digital filter graph to another digital filter graph by shifting the location of registers without moving the functionality of the circuits. Generally, it is used for reducing the delay variables count in the circuits. Because it could influence the clock period and delay variables, so it is obligatory to consider all phases into account. Additionally, it can be used to reduce switching operation which minimizes the dynamic power dissipation in circuits. A large amount of placing the component at the initial input node can reduce switching which plays a role for reducing power consumption. The main objective of retiming is to reduce the clock period. So, shifting the delay variable can be helpful in reducing the clock time period of a circuit.

### Quantitative

If the transforms digital flow graph *g* to a retimed digital flow graph $g_{r}$ then the final output or solution at the last is illustrates by a numerical quantity *r*(*v*) for all *v*. let *w*(*e*) and $w_{r}(e)$ are illustrates the weight of the edge *e* in the first digital flow graph *g* be *w*(*e*) and in the retimed digital flow graph $g_{r}$ be $w_{r}(e)$. Finally, the weight at the each edge $u\overset{e}{\rightarrow }v$ in the retimed digital flow graph $g\rightarrow g_{r}$ are evaluated by the following mathematical formulation;

4.1 $$\begin{aligned} w_{r}(e) = w(e)+r(v)-r(u) \end{aligned}$$

where *r*(*u*) and *r*(*v*) are retime output vectors.

### Clock time period (CTP) minimization

Retiming method is generally applied for the minimization of the CTP of the digital flow graphs. The least CTP for the digital flow graph, is the highest critical path computation time with no delay. The least feasible CTP, $\phi (g)$, is evaluated by the following mathematical formulation;

4.2 $$\begin{aligned} \phi (g)=max\left\{ t(p):w(p)=0 \right\} \end{aligned}$$

where $w(p)=\sum _{i=o}^{n-1}(w(e_{i}))$ and $t(p)=\sum _{i=o}^{n}(v_{i})$ are illustrates weight and computational time of the path. Further, through the following phases have been illustrated how to finds a retimining solution vector $r_{0}\mid \phi (g_{r_{0}})\leqslant \phi (g_{r})$. Here *w*(*u*, *v*) and *D*(*u*, *v*) are used in retiming method to illustrates least number of delay and maximum computational time of the path from $u\rightarrow v$.

- In a digital flow graph $u\overset{e}{\rightarrow }v$, the edge weights are evaluated by the following equations;
  4.3 $$\begin{aligned} m=I_{max}\times N \end{aligned}$$
  where *N* and $I_{max}$ are illustrates the number of nodes and maximum node execution time in digital flow graph.
  4.4 $$\begin{aligned} w'(e)=m\times w(e)-t(u) \end{aligned}$$
- In this phase, the following formulations are applied for deciding the next new shortest path in $u\overset{e}{\rightarrow }v$;
  if $$u\ne v$$
  4.5 $$\begin{aligned} D(u,v)=m\times w(u,v)-s_{u,v}+t(v) \end{aligned}$$
  where $$w(u,v)=\left\lceil \frac{s_{uv}}{m} \right\rceil $$.
  if $$u=v$$
  4.6 $$\begin{aligned} D(u,v)= t(v) \end{aligned}$$
  where $$w(u,v)=0$$.
- In this phase the CTP has evaluated by two matrices *w*(*u*, *v*) and *D*(*u*, *v*) over the following conditions of $\left\{ r\mid \phi (g_{r})\leqslant CTP \right\}$;
  **Feasibility constraint conditions;**
  4.7 $$\begin{aligned} r(u)-r(v)\le w(e) \;\; \; \forall \;\;\; u\overset{e}{\rightarrow }v \end{aligned}$$
  **Critical path (CP) constraint conditions;**
  4.8 $$\begin{aligned} r(u)-r(v)\le w(e)-1 \;\; \; \forall \;\;\; u\overset{e}{\rightarrow }v \mid D(u,v)>CTP \end{aligned}$$
  where the feasibility condition is illustrate the delay variable on every edge non-negative and similarly, the critical path condition is to forces $\phi (g)\geqslant CTP$. Similarly, if $D(u,v)>CTP$, then $w(u,v)+r(v)-r(u)\ge 1$ should satisfy for CP execution time period $\leqslant CTP$.
- In this phase, the algorithm is implemented for obtaining the retime vectors.

### Retiming for register minimization

**Figure 1:**
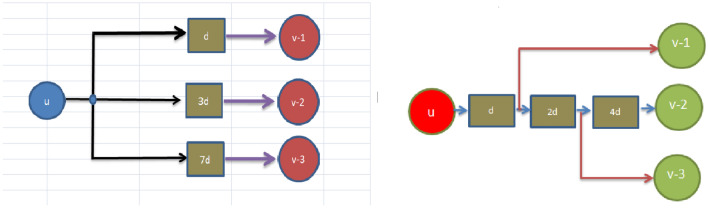
The graphs for fanout and clever implementation.

In circuit, if a single node has various output edges are connected to other nodes while the maximum delay variables needed for that output going edge is the highest delay variable of a single node. The brief details have been represented through the Fig. 1. These graphs are shows that here ’naive’ implementation shows $1+3+7=11$ registers on Fig. 1 and ’clever’ implementation shows $max(1,3,7)=7$ registers on Fig. 1. Similarly, with the help of following mathematical equation can be obtained the number of registers needed to apply the output edges of the node *v* in the retimed figure as:

4.9 $$\begin{aligned} r_{v}=\max _{v\rightarrow g_{n}}{w_r(e)} \end{aligned}$$

where $$ r_{v}$$ and $$g_{n}$$ are illustrates the total register output cost in the retimed circuit and gadget node. Here, the above function holds under three different constraints or conditions as fanout, feasibility and clock time period respectively. These conditions or subject to constraints have been illustrated through the followings;

**Fanout condition:**

4.10 $$\begin{aligned} r_v\ge w_r(e) \;\; \forall \; \; v\overset{e}{\rightarrow }\ g_{n} \end{aligned}$$

**Feasibility condition:**

4.11 $$\begin{aligned} r(u)-r(v)\leqslant w(e) \;\; \forall \;\; u\overset{e}{\rightarrow } v \end{aligned}$$

**Clock time period condition:**

4.12 $$\begin{aligned} r(u)-r(v)\leqslant w(u,v)-1 \;\; \forall \;\; vertices \; u,v\;\; st\;\; D(u,v)>CTP \end{aligned}$$

These retiming techniques would provide us with one maximal clock frequency solution. The designer would not be able to explore the entire potential solution space of the filter circuit under consideration. The entire solution space for the considered digital filters circuit is analyzed in the present evolutionary retiming algorithms and different feasible solutions are obtained. Depending on the critical path and the register count as the constraint, the designer can select any solution of his preference. Evolutionary algorithms explores the solution space by preserving all the node attributes of the digital filters graph. A framework model is generated to understand the design space for Pareto-optimal solutions. This will help the decision maker to converge at a design specification.

### High level synthesis (HLS)

HLS is the process of converting a high abstraction level description of a design to register transfer level description. This is done by using MATLAB HDL coder that generates the synthesizable VHDL or Verilog code that has been executed to HDL work flow and generates the hardware design. This save the design cycle time with the generation of synthesizable report that gives information about the improvement in the speed and complexity reduction of the design with respect to novel retiming algorithm. The Matlab HDL coder and Xilinx tool transforms the specification into a register transfer level (RTL) implementation that can synthesize into a Xilinx field programmable gate array (FPGA). HLS techniques focus on design space exploration with reduced design cycle time and allow many optimization techniques and transformations. Herein this work retiming transformations are to be incorporated into the design for performance enhancement. The design space exploration results are taken from MATLAB and Viva do HLS. The retiming solutions in MATLAB are verified in Xilinx, along with clock period. Here the problem of optimally mapping a Data Flow Graph [DFG] specification of digital filters on to FPGA architecture has been done with the help of retiming transformation. This optimality is achieved using retiming based on meta heuristic algorithm(MHA). In this work, retiming based MHA algorithms are implemented on the different structures of the digital filters using HDL coder. A synthesisable RTL is obtained from input in which the location of the registers is altered in such a way that the overall clock period reduces, thereby increasing the clock frequency. This happens due to reduction in the critical path which bounds the speed of the design. Further, intelligent placement of registers is implemented that minimized the area. It is observed that the operating clock frequency in the digital filter can be increased to a great extent after novel approach.

Clock period and number of registers are considered as the optimization requirement in the present work. Using MHA, multiple retimed solutions are generated with high speed and different output register counts. Depending on the area constraint, user can choose the retiming solution with particular register counts. The MHA approach helps to find all possible retimed solutions and obtaining synthesizable HDL of the considered filter. For this, model is designed which automatically generates the synthesizable HDL of the considered filter. Again the choice of HDL (VHDL or Verilog) can be given by the user. This optimization environment reduces lot of design cycle time for the considered digital filters. The designer can choose any solution depending on the time units for critical path and the number of registers. With the designed environment, the designer can choose the required solution and can get the synthesizable HDL.

## Modified SChoA version for high level synthesis of digital filters

The sequential and recursive filtering of circuits is a complex optimization problem for the recent demand of the technology. The scientists are trying to solve this issue with the help of new one presented algorithms. However, each and every algorithm is not able to tackle these complex problems while these methods can be trapped in these types of complex issues. So, robust methods are required to resolve the complexity of these functions. According to present demand we are trying to present the new modified version SChoA for handling these complexities. This modified version is the mixture of two population based algorithms such as chimp and sine cosine methods. These trigonometric functions have been applied over the position update equation of the chimp optimizer for enhancing the exploitation phase. In addition, this enhancement, is developed for tackling various complex issues as slow diversity, premature convergence and slow convergence speed etc. Here all algorithms have been applied to solve the retiming issue as well as clock time period and area are the given constraints. Let *v* and $e_{ij}$ illustrate the set of nodes and edges, where each edge is linked amid (*i*, *j*) vertices like $i \ne j$. In data flow graphs, delay is denoted the registers while linking nodes by an edge illustrated by weight vector $w_{ij}$. The following three quantities are evaluates is the main prospectus of this implementation.

- the least number of registers amid two paths on any path.
- the least execution time required amid two paths on any path.
- the high-level synthesis (HLS) of datapaths in digital filters.

In this stage, we are explain the implementation steps of the proposed method, that how to insert these digital filter functions with the proposed algorithm.

### System inequality

The main task of the proposed method that is minimize the fitness function *t*(*l*, *m*, *r*) for $t:g\overset{e}{\rightarrow }g_{r}$. The subject to constraints as followings;

- *l*: critical path
- *m*: Registers
- *r*: Total time required for evaluation

Also, find the output area of this issue to get all global retimed outputs. The many retimed outputs *m* created required to have;

5.1 $$\begin{aligned} l= & {} \left\{ l_{1}\ge 0,l_{2}\ge 0,\dots , l_{m}\ge 0 \right\} \end{aligned}$$

5.2 $$\begin{aligned} m= & {} \left\{ m_{1}\ge 0,m_{2}\ge 0,\dots , m_{m}\ge 0 \right\} \end{aligned}$$

Here the following cost function has been applied for calculating the global output given by $T(x)=\left\{ t_1(x),t_2(x),\dots , t_m(x) \right\}$, where *m* illustrates the global outputs. The following mathematical formulation has been consider for optimization are

5.3 $$\begin{aligned} T(x)=min\left\{ \sum _{i=0}^{m}l_i \sum _{j=0}^{n}m_j\right\} \end{aligned}$$

### Parameters

In this research the various population based methods have been runned parallel for fair comparison. During this implementation has been used thirty search members and five hundred maximum number of generations.

### Initialization

In the search domain the decision variable values of the given function are the same as the location of the search members of the population. The location of the each member of the crowd is assign as the following mathematical formulation (5.4);

5.4 $$\begin{aligned} X= \begin{bmatrix} x_{1,1} &{} x_{1,2} &{}, ... , &{} x_{1,d}\\ x_{2,1} &{} x_{2,2} &{}, ... , &{} x_{2,d}\\ \vdots &{} \vdots &{} \ddots &{} \vdots \\ x_{n,1} &{} x_{n,2} &{}, ... , &{} x_{n,d}\\ \end{bmatrix} \end{aligned}$$

Where *n* and *d* are illustrates the total number of search member in the search domain and dimension respectively. The fitness values of the each search member can be evaluate by the following mathematical Eq. (5.5);

5.5 $$\begin{aligned} FX=\begin{bmatrix} fx_{1} \\ fx_{2} \\ \vdots \\ fx_{n} \\ \end{bmatrix} \end{aligned}$$

where *n* and $fc_{i}$ are illustrates the total of number search members and fitness outputs of the $i^{th}$ member. Similarly, another two matrices can be formulate for the last node *z* (or target) of the graph by the following mathematical equations;

5.6 $$\begin{aligned} Z= & {} \begin{bmatrix} z_{1,1} &{} z_{1,2} &{}, ... , &{} z_{1,d}\\ z_{2,1} &{} z_{2,2} &{}, ... , &{} z_{2,d}\\ \vdots &{} \vdots &{} \ddots &{} \vdots \\ z_{n,1} &{} z_{n,2} &{}, ... , &{} z_{n,d}\\ \end{bmatrix} \end{aligned}$$

5.7 $$\begin{aligned} FZ= & {} \begin{bmatrix} fz_{1} \\ fz_{2} \\ \vdots \\ fz_{n} \\ \end{bmatrix} \end{aligned}$$

where *n*, *d* and $fz_{i}$ are illustrates the number of nodes, dimension of the function and the fitness value of the *i*th node.

### New location and distance b/w nodes

In this stage, the position of the search member in the search domain and distance of the given node is calculated by the following Eqs. (5.8)–(5.12), here these mathematical equations are illustrates the new location of the variable of the search member. Further, the Eqs. (3.1)–(3.5), are illustrates the distance b/w the position of the variable of the search member and the position of decision variables of the target.

5.8 $$\begin{aligned} r_2= & {} (2 \pi )\times rand \end{aligned}$$

5.9 $$\begin{aligned} x^*_1= & {} a_{attacker}-cos(r_2)\times a_1.d_{attacker} \end{aligned}$$

5.10 $$\begin{aligned} x^*_2= & {} a_{barrier}-sin(r_2)\times a_2.d_{barrier} \end{aligned}$$

5.11 $$\begin{aligned} x^*_3= & {} a_{chaser}-cos(r_2)\times a_3.d_{chaser} \end{aligned}$$

5.12 $$\begin{aligned} x^*_4= & {} a_{driver}-sin(r_2)\times a_4.d_{driver} \end{aligned}$$

For modified the location of the each member of the crowd in every generations with the aims of the improving the extractability of the proposed method has applied the following mathematical Eq. (5.13).

5.13 $$\begin{aligned} C_{n_s}= \left( n_s-G \right) \times \frac{n_s}{M_{G}} \end{aligned}$$

where $n_s$,*G* and $M_{G}$ are illustrates the number of search member, current iteration and maximum generations. Therefore, the search member of the crowd update their locations during the search process in the search area with respect to the target in the last generations.

### Leader search member position

The leader search member position modified by the following mathematical Eq. (5.14);

5.14 $$\begin{aligned} x^*_{n+1}=\frac{x^*_1+x^*_2+x^*_3+x^*_4}{4} \end{aligned}$$

Lastly the following Eq. (5.15) has been used for updating the position of new path in the retimed data flow graph;

5.15 $$\begin{aligned} a_{s}\left( n+1 \right) =\left\{ \begin{matrix} a_{t}\left( n \right) -x.d,&{} if &{} \phi <0.5 \\ chaotic_{value}&{} if &{} \phi >0.5 \end{matrix}\right. \end{aligned}$$

where $a_{s}$ and $a_{t}$ illustrate the updated location of new path by search member and location of the last node or target position.

### Fitness function

The following fitness function^71^ has been applied for testing the best solution of the retiming problem with least cost path over the following subject to three different properties;

5.16 $$\begin{aligned} F= & {} \left[ \frac{1}{(R+M)} \right] \end{aligned}$$

5.17 $$\begin{aligned} R= & {} \frac{1}{L_{max}} \end{aligned}$$

where *R*, *M* and $L_{max}$ are illustrates the critical path, number of registers and longest path of the filter.

#### Subject to constraints

Here subject to constraints are hold over three different properties such as;

- **Prop-1:** The weight (*w*) of the graph must be capable of rewriting as the weight (*w*) of the edge of the original graph, with the retimed value of each node. This can be mathematical formulate in the following form;
  5.18 $$\begin{aligned} w_{z}^{v}=w_{z_0\rightarrow z_1}^{v}+w_{z_1\rightarrow z_2}^{v}+\dots +w_{z_{k-1}\rightarrow z_k}^{v}=\sum _{i=0}^{j=k-1} w_{z_{j}\rightarrow z_{j+1}}^{v} \end{aligned}$$
  where $z_0$ and $z_k$ illustrate the start and end nodes in *z* of the retimed graph. The above mathematical formulation can be rewritten in the following form;
  5.19 $$\begin{aligned} w_{z}^{v}=\sum _{i=0}^{j=k-1} w_{z_{j} \rightarrow z_{j+1}}^{v}+v_{r_{i}}-v_{r_{i}}=w_{z}+\sum _{i=1}^{j} v_{r_{i}}+\sum _{i=0}^{j-1} v_{r_{i}} \end{aligned}$$
  where *v* is represent the retimed vector.
- **Prop-2**: In a retimed data flow graph (DFG), the weight (*w*) of the blocked way including the loop bound and repeated filing of this graph, should not exchange. In DFG the loop of a cycle is determined by the total time needed to run that particular circle. This is evaluated by summing up all nodes in the graph or cycle. And the generation bound illustrates the highest loop bound of every cycle in this graph.
- **Prop-3**: The initial and last node of the edge in the data flow graph way would remain the same. This can be evaluated through the following mathematical equation;
  5.20 $$\begin{aligned} w_{u\rightarrow v}^{v}=w_{u\rightarrow v} \end{aligned}$$
  If any output not fulfil these conditions would be the global output value with a given cost fitness function. We may penalize retimed output that do not fulfil the conditions with the value of the penalty that discharges these individuals during the selection process. The cost of the given function is evaluated in terms of number of registers and critical path after retiming. So, it is calculated by the above fitness function (5.16).

### Stopping condition

Lastly, the stopping criteria has been applied for updating the new one best path for the search member in the data flow graph. This process repeated again and again until it satisfies the criteria of prevention for example it reaches the highest generations or the output is earliest found.

### Pseudocode of the proposed algorithm

The pseudocode of modified SChoA version is illustrated in Algorithm 2.
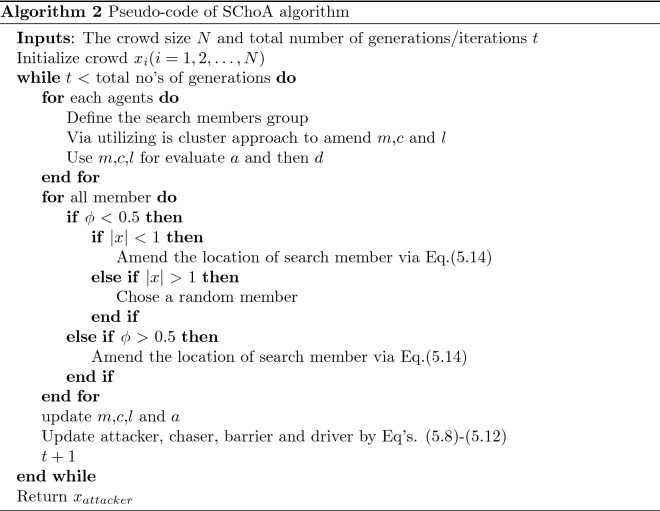


## Results analysis and discussion of 23-standard test suites

For evaluating the performance of the enhanced version has been used 23-standard test suites. These functions have been divided into three different phases such as 07-uni-modal, 06-multi-modal and 10-fixed dimension multi-modal test suites respectively. These test suites have been reported in the appendix table S1. The convergence performance and robustness of the enhanced algorithm have been compared with recent powerful optimizers such as Chimp, TACPSO, SCA and MPSO etc. Further, the analysis and discussions of the results have been illustrated in details in the following sub-sections:

### Constant setting and standard test suites

All the evolutionary methods have been coded in the Matlab R2018a during the implementation. And algorithms have been runned on the system Intel(R) Core(TM) i3-8130U, RAM 8GB and Win 10. In these experiments have been applied different constant settings such as 30-number of search agents and 500- number of iterations respectively. It is constantly advantageous to use a standard test suite with dissimilar features to suitably and assertively verify the robustness of MA’s on altered standard test suites and compare it with recent MA’s. The diversity of these test suites permits detecting and analyzing the capability of the proposed method from dissimilar standpoints. In uni-modal test suites have only one global optimum with no local optima. Generally, these test suites are highly suitable for comparing the convergence and exploitative ability of the MA’s. Additionally, the multi-modal and fixed dimension multi-modal test suites face the survival of numerous local optimum outputs and more than one global optimum.

Here, the 23-test suites have been used to validate the robustness and efficiency of the enhanced version compared to with others recent MA’s. These test suites have been divided into three categories such as uni-model, multi-modal and fixed dimension multi-modal functions. These test suites have been illustrated through appendix table S1.

### Discussion

The performance of the algorithms have been tested on 23-test suites and experimental results are illustrated in the table. The robustness of the algorithms have been verified in terms of best minimum cost, maximum cost, average and standard deviation etc. Here the least and maximum cost of the fitness test suites illustrates the best performance of the evolutionary algorithms. And the statistical outputs have also been used for testing the robustness of the algorithms. All these results have been computed at the last iteration for every evolutionary algorithm on every test suite to get the best global optima solution, to compare meaningful best outputs.

#### Assessment of exploitation capability

Firstly, the performance of the evolutionary algorithms have been tested on uni-modal test suites (F1–F7). Generally, these test suites are applied to evaluate the exploitation phase. The results of algorithms on these test suites have been illustrated through Tables 1, 2, 3 and 4. All the results of these tables gives strong evidence that the enhanced algorithm has been able to provide the better exploitation ability as compared to others. As mentioned earlier, these standard test suites are most suitable for these functions. Experimental outputs prove that the SChoA algorithm is highly functional. Furthermore, these experimental solutions prove that the proposed strategy can be highly effective and robust in giving the accurate and best optima for the high complex space test suites as compared to others.

**Table 1:** The performance of algorithms on the 23-test suites.

Test suites	SChoA				Chimp			
F1–23	Min	Max	Mean	S.D	Min	Max	Mean	S.D
F1	**3.44E−33**	**7.27E+04**	**2.41E+03**	**3.65E+03**	1.19E−05	6.04E+04	3.36E+04	2.79E+04
F2	**2.93E−20**	**5.92E+12**	**2.38E+08**	**1.74E+09**	3.77E−05	3.47E+12	6.41E+11	1.28E+12
F3	**3.64E−08**	**1.38E+05**	**1.04E+04**	**2.11E+04**	4.30E+01	1.27E+05	5.58E+04	5.17E+04
F4	**1.77E−11**	**9.56E+01**	**1.08E+01**	**2.36E+01**	5.64E−02	8.83E+01	5.41E+01	3.77E+01
F5	**1.86E+01**	**3.10E+08**	**7.06E+06**	**3.36E+07**	2.90E+01	2.99E+08	1.45E+08	1.35E+08
F6	**1.20E−03**	**7.90E+04**	**1.02E+03**	**2.29E+03**	3.72E+00	6.57E+04	3.30E+04	2.91E+04
F7	**3.91E−04**	**1.78E+02**	**1.16E−02**	**1.66E−01**	1.80E−03	1.02E+02	5.69E+01	4.70E+01
F8	**1.04E+00**	**4.98E+02**	**3.64E−01**	**1.88E+00**	6.86E+00	4.45E+02	2.64E+02	1.97E+02
F9	**0.00E+00**	**4.88E+02**	**2.75E+00**	**1.50E+00**	5.78E+00	4.24E+02	2.75E+02	1.84E+02
F10	**2.22E−14**	**2.09E+01**	**2.42E+00**	**5.54E+00**	2.00E+01	2.07E+01	2.00E+01	4.46E−02
F11	**0.00E+00**	**6.00E+02**	**1.09E+01**	**2.51E+01**	1.14E−05	5.53E+02	3.30E+02	2.98E+02
F12	**0.00E+00**	**7.14E+08**	**1.07E+07**	**6.47E+07**	3.06E−01	4.99E+08	2.80E+08	2.31E+08
F13	**9.99E−01**	**6.40E+01**	**1.07E+00**	**2.05E+00**	9.98E−01	1.08E+01	2.91E+00	3.02E+00
F14	$-$**1.03E+00**	**0.00E+00**	$-$**1.02E+00**	**2.40E−03**	$-$1.03E+00	$-$2.51E−01	$-$1.00E+00	1.23E−01
F15	**6.80E−04**	**1.85E−01**	**2.00E−03**	**9.70E−03**	1.30E−03	8.09E−02	2.10E−03	5.80E−03
F16	**3.98E−01**	**2.53E+00**	**4.10E−01**	**9.70E−02**	3.98E−01	2.12E+00	4.25E−01	8.30E−02
F17	**3.98E−01**	**4.72E−01**	**4.00E−01**	**1.08E−02**	3.99E−01	1.33E−01	4.01E−01	9.01E−02
F18	**3.00E+00**	**8.22E+01**	**3.06E+00**	**6.17E−01**	3.00E+00	4.14E+01	3.21E+00	1.79E+00
F19	$-$**3.86E+00**	**0.00E+00**	$-$**3.83E+00**	**5.46E−02**	$-$3.85E+00	$-$2.25E+00	$-$3.77E+00	2.90E−01
F20	$-$**3.82E+00**	$-$**1.16E+00**	$-$**3.63E+00**	**2.19E−01**	$-$3.13E+00	$-$2.42E+00	$-$2.76E+00	2.89E−01
F21	$-$**1.01E+01**	$-$**1.16E+00**	$-$**4.80E+00**	**2.72E−02**	$-$8.82E−01	$-$7.64E−01	$-$8.69E−01	2.79E−02
F22	$-$**1.04E+01**	$-$**5.20E−01**	$-$**4.88E+00**	**8.38E−02**	$-$5.00E+00	$-$5.68E−01	$-$3.95E+00	1.20E+00
F23	$-$**1.04E+01**	$-$**2.10E−01**	$-$**4.32E+00**	**8.38E−02**	$-$5.00E+00	$-$6.68E−01	$-$3.95E+00	1.20E+00

The bold values show the best solutions for problems.

#### Capability assessment

Further, the performance of the evolutionary algorithms have been verified on multi-modal ($F_8$–$F_{13}$) and fixed dimension multi-modal ($F_{14}$–$F_{23}$) test suites. The all results of algorithms on these test suites have been illustrated through Tables 1, 2, 3 and 4. These test suites have many local and the number of decision variables increases exponentially with the size of the test suite compared to the uni-modal test suite. Generally, these test suites are used to assess the exploration ability and suitability of the evolutionary algorithms. All results of tables, shows that the enhanced strategy achieves a higher detection ability and superior exploitation ability.

#### Accuracy

In this subsection, the average scores obtained by the evolutionary algorithms on 23-test suites have been discussed briefly in the Table 2. The average values have been divided into two categories like worst average score (*W*) and best average score (*B*) etc. Generally, the least average score denotes the accuracy of the evolutionary algorithms for the best outputs. In the Table 2, we can see easily that the proposed strategy is able to find the best optima solutions in at least average scores for the maximum standard test suites in the highly complex space. Hence these results give strong evidence that the proposed strategy can find the accurate solutions for the highly complex test suites as compared to others.

**Table 2:** Comparison of mean outputs of evolutionary algorithms on 23-standard test suites.

Test suites	SChoA	Chimp	TACPSO	SCA	MPSO
F1	B	W	W	W	W
F2	B	W	W	W	W
F3	B	W	W	W	W
F4	B	W	W	W	W
F5	B	W	W	W	W
F6	B	W	W	W	W
F7	B	W	W	W	W
F8	B	W	W	W	W
F9	B	W	W	W	W
F10	B	W	W	W	W
F11	B	W	W	W	W
F12	W	W	B	W	W
F13	B	W	W	W	W
F14	W	W	W	W	B
F15	B	W	W	W	B
F16	W	W	W	W	B
F17	B	W	B	W	W
F18	B	W	W	W	B
F19	W	W	W	W	B
F20	B	W	W	W	W
F21	W	W	W	W	B
F22	B	W	W	W	W
F23	W	W	W	W	B

#### Stability

**Figure 2:**
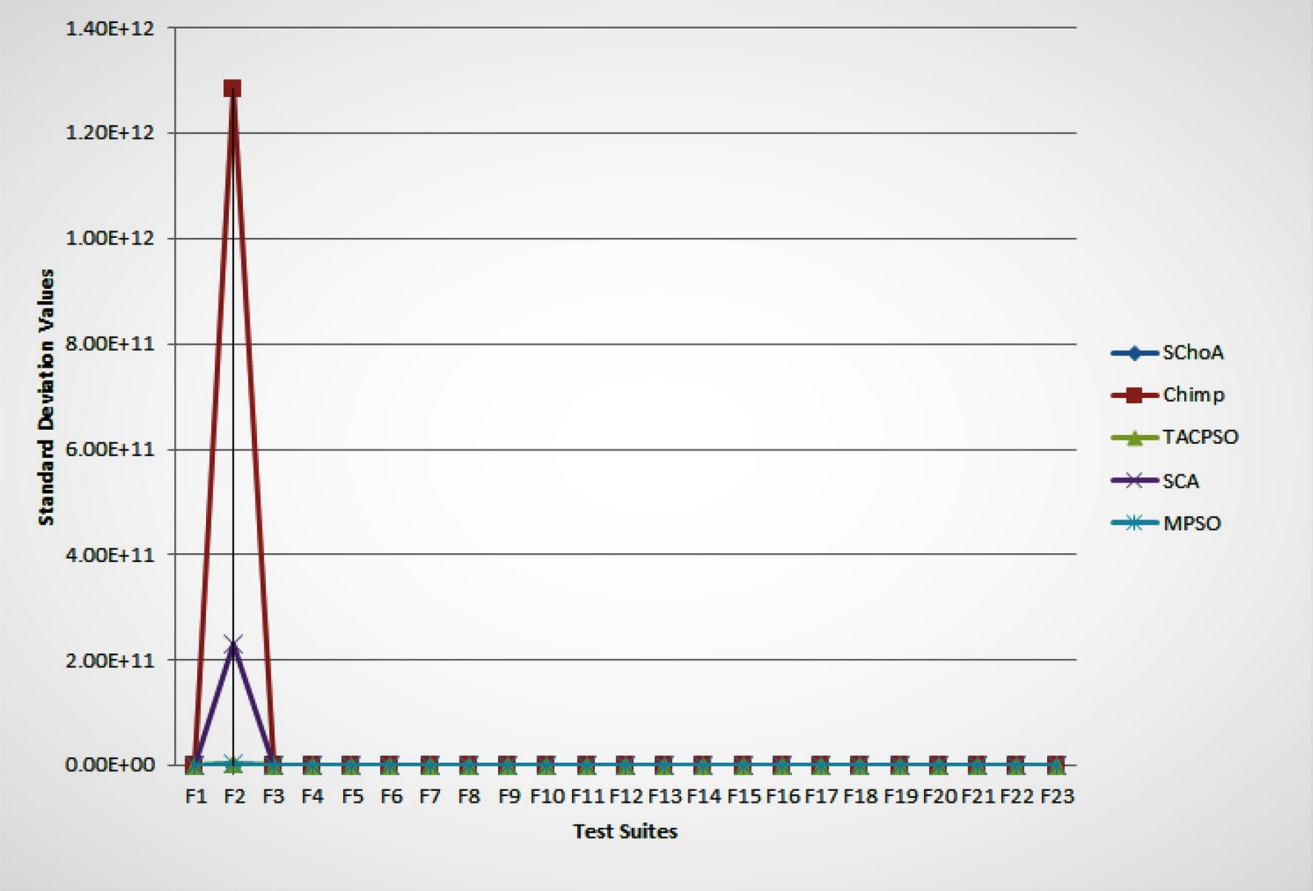
The standard deviation (SD) values of evolutionary algorithms on 23-standard test suites.

The standard deviation is used to verify the solution stability of the evolutionary algorithms. In this phase, the performance of the evolutionary algorithms have been discussed on the behalf of statistically. The standard deviation values obtained by the evolutionary algorithms on the 23-test suites have been plotted through Fig. 2. In this graph, we can see easily that the standard deviation values of the proposed strategy on 23-test suites are near to 0, it means that the proposed strategy is stable on the test suites that were performed. Additionally, the least standard score shows the best convergence performance of the evolutionary algorithms. Hence, here, it can be concluded that the proposed methodology can able to fastly trap the best global optima solution in the search space as compared to others.

**Table 3:** The performance of algorithms on the 23-test suites.

Test suites	TACPSO				SCA			
F1–23	Min	Max	Mean	S.D	Min	Max	Mean	S.D
F1	1.00E−02	6.17E+04	3.58E+03	5.30E+03	5.57E+01	6.43E+04	1.31E+04	2.27E+04
F2	1.19E+00	7.34E+10	2.94E+08	4.63E+09	9.60E−03	5.16E+12	1.03E+10	2.31E+11
F3	7.72E+02	1.08E+05	7.64E+04	2.99E+04	3.49E+03	1.20E+05	4.82E+04	4.16E+04
F4	1.05E+01	8.30E+01	2.09E+01	1.16E+01	3.19E+01	9.17E+01	6.65E+01	2.67E+01
F5	2.76E+01	1.93E+08	8.67E+06	4.31E+07	1.80E+05	1.52E+08	7.34E+07	7.44E+07
F6	2.24E−01	5.93E+04	1.58E+03	4.66E+03	4.28E+00	6.84E+04	7.92E+03	1.60E+04
F7	1.18E−01	1.55E+02	1.66E+00	8.94E+00	1.02E+00	7.51E+01	1.82E+01	2.24E+01
F8	7.39E+01	4.39E+02	1.34E+02	7.89E+01	7.06E+01	4.38E+02	1.81E+02	1.05E+02
F9	8.36E+01	4.29E+02	1.42E+02	7.66E+01	0.00E+00	4.60E+02	1.60E+02	1.42E+02
F10	4.26E+00	2.08E+01	6.71E+00	4.00E+00	9.60E−01	2.06E+01	8.85E+00	8.04E+00
F11	1.27E−01	5.94E+02	1.77E+01	4.79E+01	1.53E+00	4.14E+02	1.29E+02	1.52E+02
F12	1.60E+00	7.01E+08	2.68E+06	3.53E+07	1.37E+01	7.02E+08	3.40E+08	3.46E+08
F13	9.98E−01	3.81E+01	2.37E+00	2.05E+01	9.98E−01	1.37E+01	2.02E+00	2.72E+00
F14	$-$1.02E+00	$-$8.01E−01	$-$9.07E−01	1.11E−01	$-$1.03E+00	0.00E+00	$-$1.02E+00	6.24E−02
F15	7.83E−04	1.65E−01	7.83E−04	0.00E+00	1.50E−03	3.70E−02	2.20E−03	4.20E−03
F16	3.98E−01	4.49E−01	4.00E−01	7.60E−03	4.02E−01	1.87E+00	4.78E−01	2.61E−01
F17	3.98E−01	3.14E−01	4.00E−01	1.84E−02	3.99E−01	1.34E+00	4.23E−01	9.71E−02
F18	3.00E+00	7.72E+01	3.22E+00	3.78E+00	3.00E+00	5.89E+01	3.23E+00	2.78E+00
F19	$-$3.86E+00	$-$3.06E+00	$-$3.85E+00	8.01E−02	$-$3.82E+00	0.00E+00	$-$3.79E+00	1.71E−01
F20	$-$2.81E+00	$-$1.40E+00	$-$2.07E+00	5.98E−01	$-$2.80E+00	$-$2.80E+00	$-$2.60E+00	3.69E−01
F21	$-$4.61E+00	$-$3.38E−01	$-$9.41E−01	1.25E+00	$-$4.97E−01	$-$4.97E−01	$-$4.93E−01	8.30E−01
F22	$-$9.12E−01	$-$3.52E−01	$-$6.76E−01	2.15E−01	$-$4.83E+00	$-$4.83E+00	$-$2.79E+00	1.74E+00
F23	$-$9.12E−01	$-$3.52E−01	$-$6.76E−01	2.15E−01	$-$4.83E+00	$-$2.98E+00	$-$2.79E+00	1.74E+00

**Table 4:** The performance of algorithms on the 23-test suites.

Test suites	MPSO			
F1–23	Min	Max	Mean	S.D.
F1	3.43E−01	6.50E+04	9.43E+03	1.38E+04
F2	6.24E−01	1.20E+09	3.45E+08	5.42E+09
F3	3.36E+04	2.19E+05	5.70E+04	2.48E+04
F4	1.53E+01	7.13E+01	3.92E+01	1.67E+01
F5	5.94E+02	2.57E+08	2.32E+07	3.73E+07
F6	5.10E−02	7.35E+04	1.22E+04	1.76E+04
F7	5.95E−02	1.09E+02	1.24E+01	1.87E+01
F8	1.19E+02	4.36E+02	2.30E+02	1.03E+02
F9	1.26E+02	4.60E+02	2.34E+02	1.03E+02
F10	1.51E+00	2.08E+01	9.55E+00	7.26E+00
F11	1.99E−01	5.69E+02	7.49E+01	1.11E+02
F12	5.08E+00	5.28E+08	7.48E+07	1.15E+08
F13	9.98E−01	1.80E+01	1.92E+00	2.78E+00
F14	− 1.03E+00	2.01E−01	− 1.02E+00	5.98E−02
F15	7.83E−04	1.59E−01	2.00E−03	1.03E−02
F16	3.98E−01	4.00E−01	3.98E−01	4.62E−04
F17	3.98E−01	3.00E−01	4.01E−01	1.67E−02
F18	3.00E+00	5.31E+01	3.14E+00	2.28E+00
F19	− 3.82E+00	− 2.99E+00	− 3.86E+00	5.72E−02
F20	− 3.20E+00	− 1.62E+00	− 3.17E+00	1.18E−01
F21	− 5.10E+00	− 3.44E−01	− 4.93E+00	5.30E−01
F22	− 5.13E+00	− 7.21E−01	− 4.12E+00	6.30E−01
F23	− 5.13E+00	− 7.21E−01	− 4.87E+00	6.30E−01

#### Convergence performance

**Figure 3:**
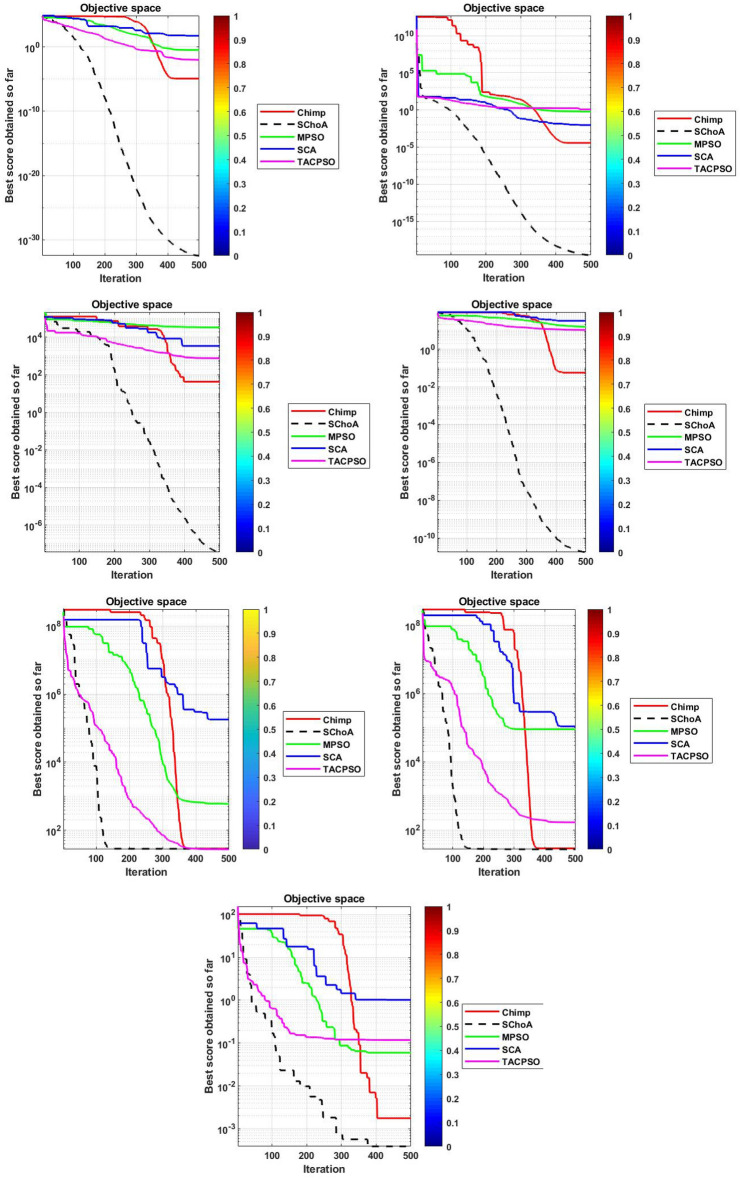
The convergence graph of algorithms on 07-Uni-modal test suites.

**Figure 4:**
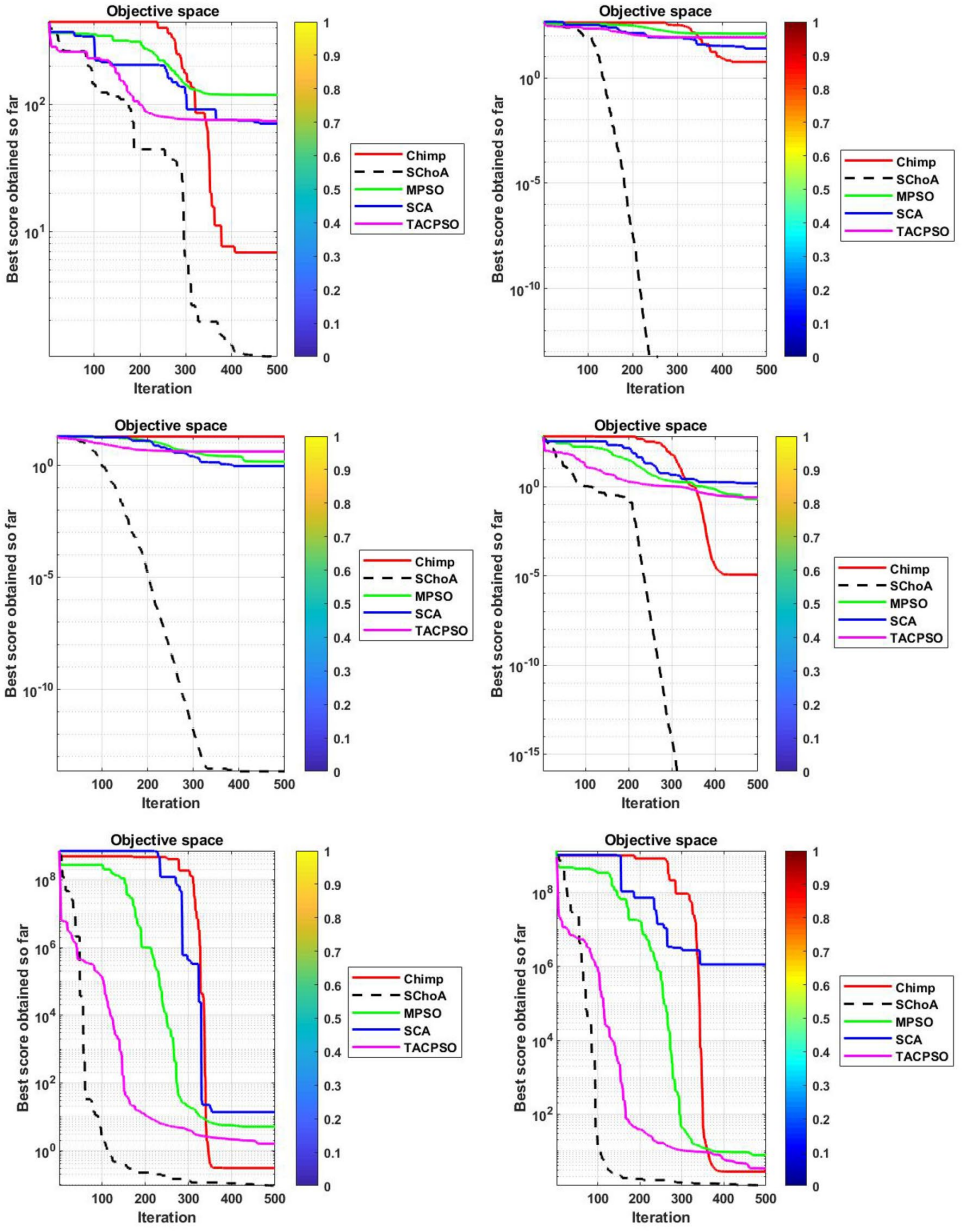
The convergence graph of algorithms on 06-Multi-modal test suites.

**Figure 5:**
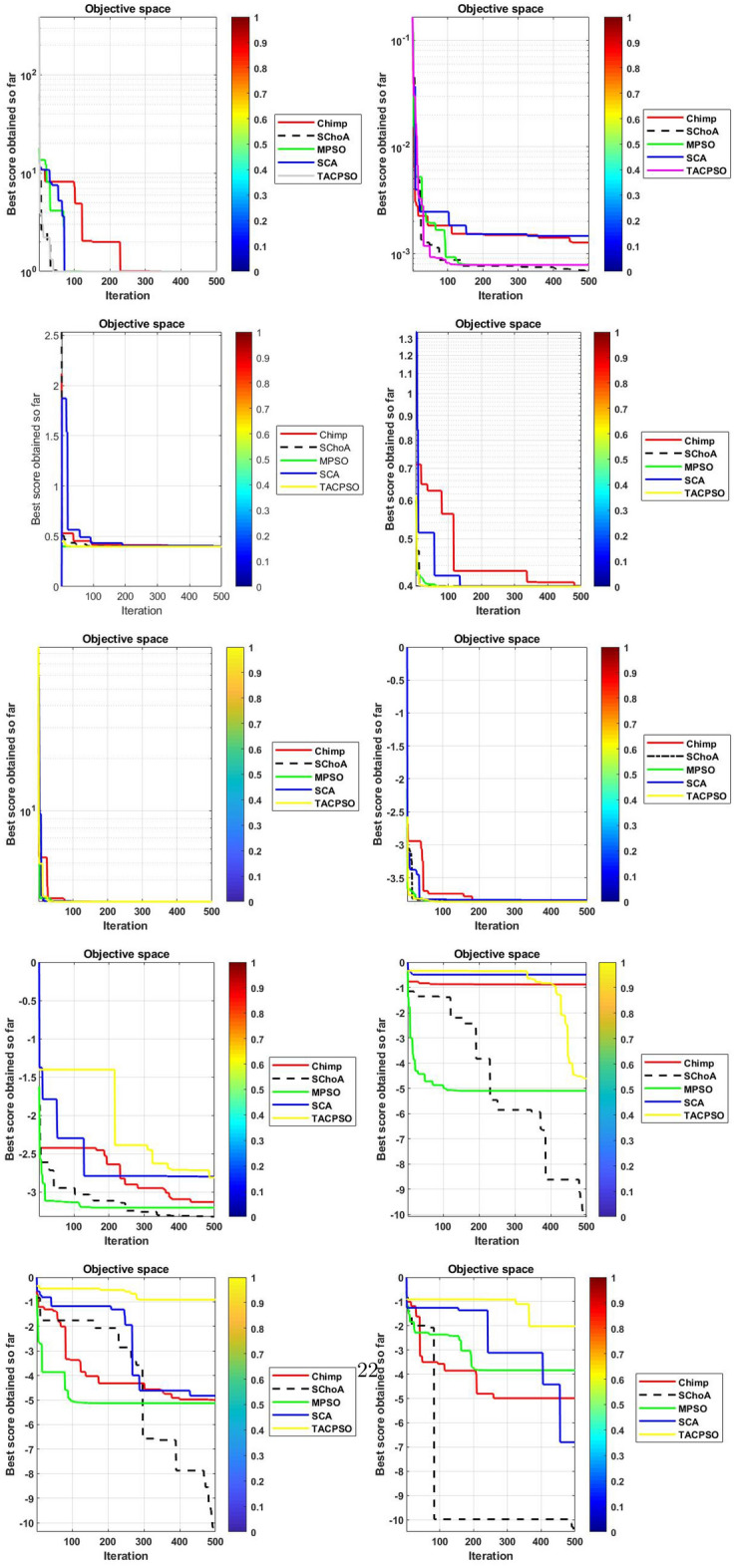
The convergence graph of algorithms on 10-Fixed dimension multi-modal test suites.

In this phase we are discussing the convergence performance of the evolutionary algorithms on 23-test suites. All these graphs have been plotted through Figs. 3, 4 and 5. In this graph the x-axis and y-axis are denoted the number of iterations and best solutions respectively. These graphs show the evolutionary algorithm how much takes a number of iterations or time for finding the best score in the search space during the search process. Additionally, as per Berg et al.^72^, this behavior can assure that evolutionary methods ultimately converge to a point and are found locally.

So, being these reasons, we can discuss and as per reasoned to the proposed methodology. The search members move from high score to low scores, so with the assumption of growth in proposed methodology, the overall chimps and their fitness are improved during the iterations. With this methodology, we save the best score for finding the next one best score and this value helps the search member during the search process in the search space for searching the next best score. On the basis of these graphs, we can conclude that the proposed strategy is able to trap the best score in least numbers of generations or time as compared to others. And could be capable of resolving very complex issues easily.

Here, the proposed method have demonstrated their efficiency and capability over traditional optimizers for generating the topology, rules and optimal parameters that deliver the superior classification performance with concerning to the quality of the global result, avoiding local minima and computational cost. So,the proposed method can be more helpful for addressing the complex domain issues and new Challenges.

## Simulations and results of digital filters

In this work, an enhanced version of chimp optimizer have been applied on 14- different data filters such as DF-FIR, LATFIR, CASFIR, PARFIR, TDFFIR, DF1IIR, TDF1IIR, TDF2IIR, DF2IIR, CASIIR, SLATIIR, DLATIIR, PARIIR and LADIIR for evaluating the several outputs. Under designing the complex filters high level synthesis is a large paramount stage for that. Generally, the high-level optimization methodology has been applied for reducing the designing period time at the lower levels, leading to superior circuit indices^73^. In this work is synthesized using MATLAB HDL coder and Viva do HLS. All the benchmarks are synthesized on the Virtex family in term of maximum usable frequency, Critical path delay and no of slices utilized in term of flipflps, LUTs, no of DSP slices etc. Other HLS tool available in the market are Stratus HLS from Cadence, HDL coder from MATLAB, Intel FPGA, Viva do HLS from Xilinx.

During implementation, Matlab HDL coder is preferred. It generates synthesizable VHDL code from MATLAb , Simulink models. The HDL coder provide the workflow advisor that automates the program that be used for programming for Xilinx. The Xilinx High-Level Synthesis (HLS) compiler provides a programming environment similar to those available for application development on both standard and specialized processors. The programming model of an FPGA was centered on register-transfer level (RTL) descriptions which illustrates how the programming model difference affects implementation time and achievable performance for different computation platforms. During this methodology, the number of registers can exceed which may be the constraints to the designer. Here, we are trying to present the superior quality of solution for these issues. The various recent algorithms and modified algorithms have been applied for verifying the accuracy of the solutions of this issue. The matlab code of all the algorithms have been runned over the system with Intel (R) Core (TM) i3-8130 U processor and 8GM of RAM. In this implementation the various parameter values applied like number of search agents (30) and number of 500 iterations respectively.

**Figure 6:**
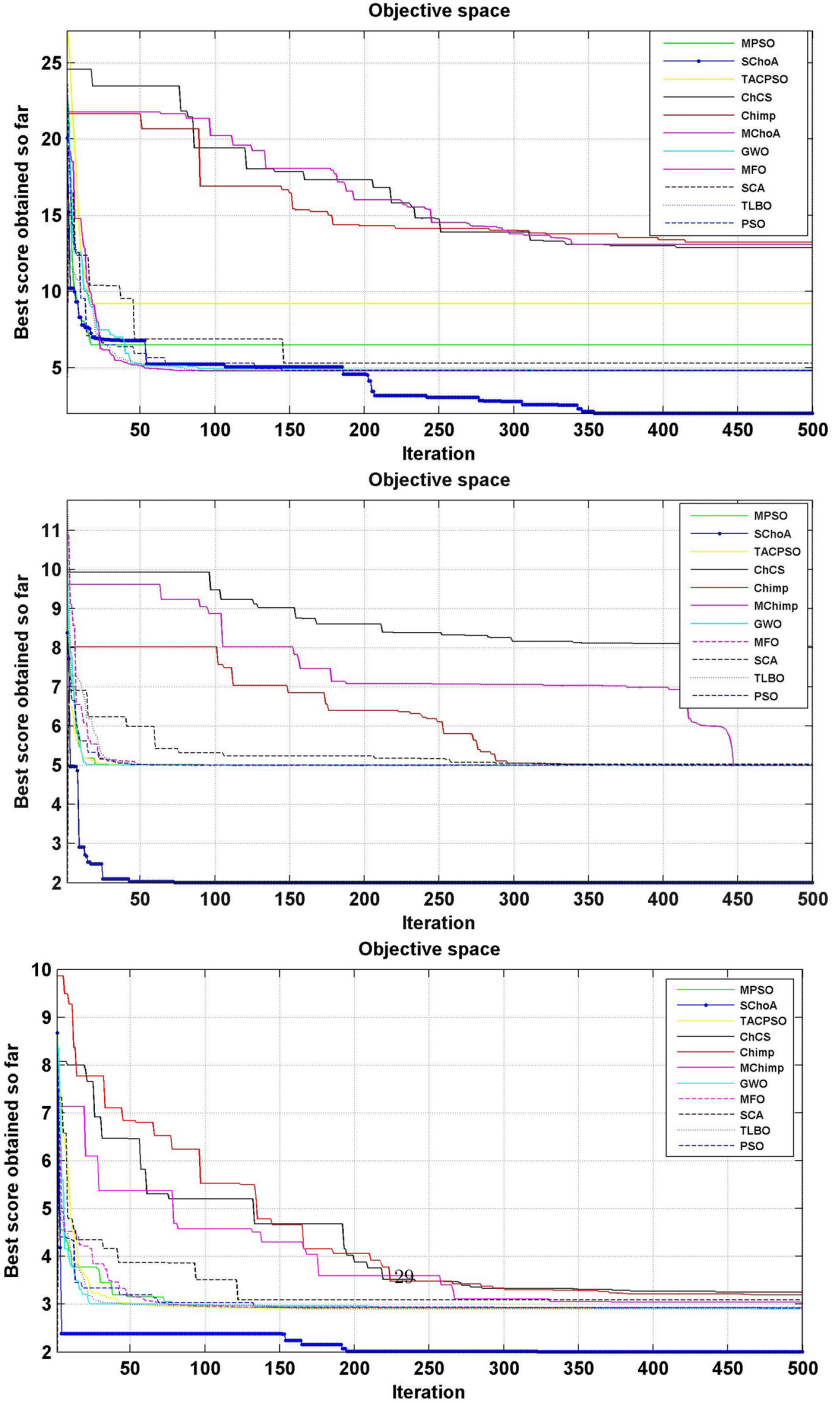
The convergence graph of algorithms on single-objective DF-FIR, LATFIR and CASFIR digital filters.

**Figure 7:**
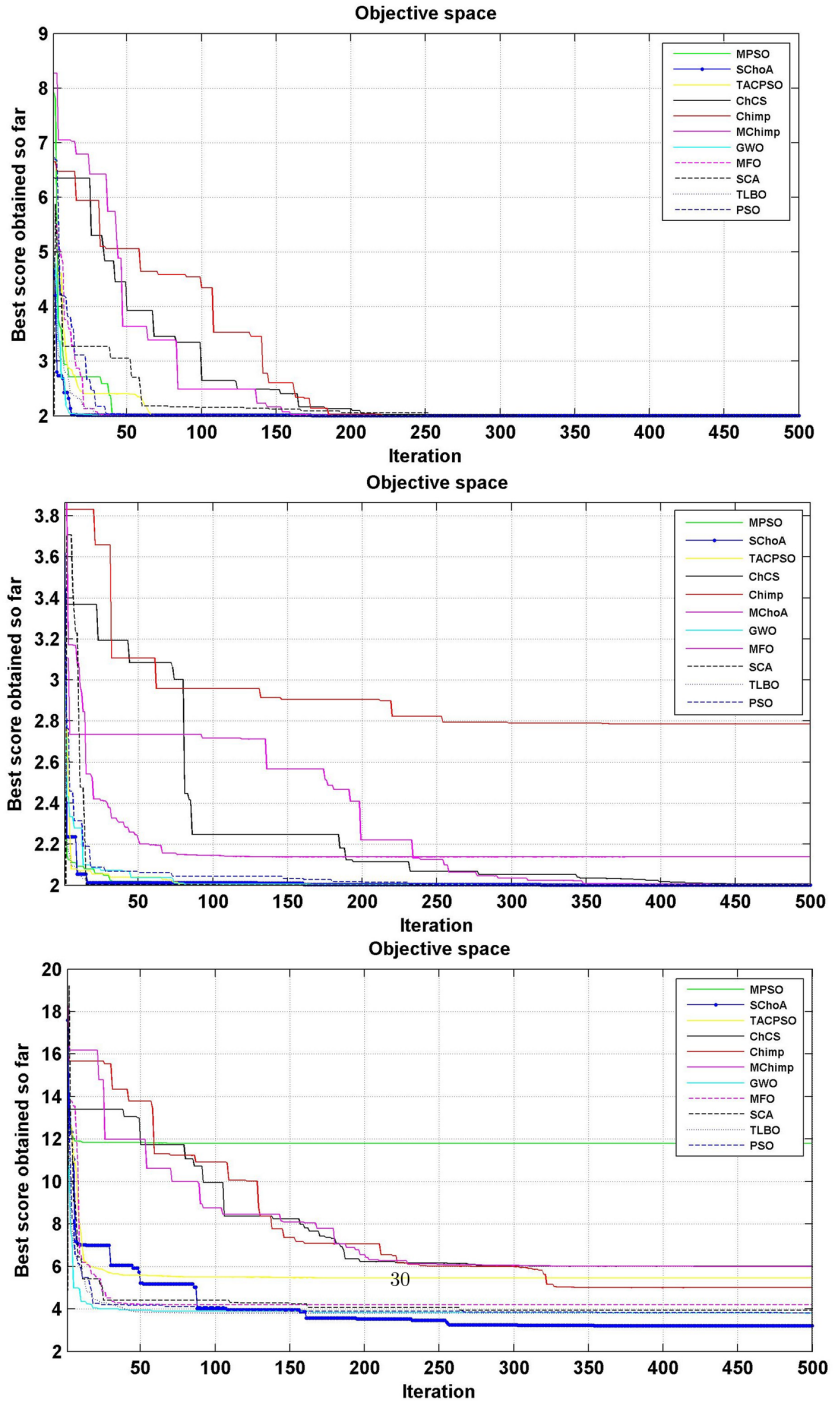
The convergence graph of algorithms on single-objective PARFIR, TDFFIR and DF1IIR digital filters.

**Figure 8:**
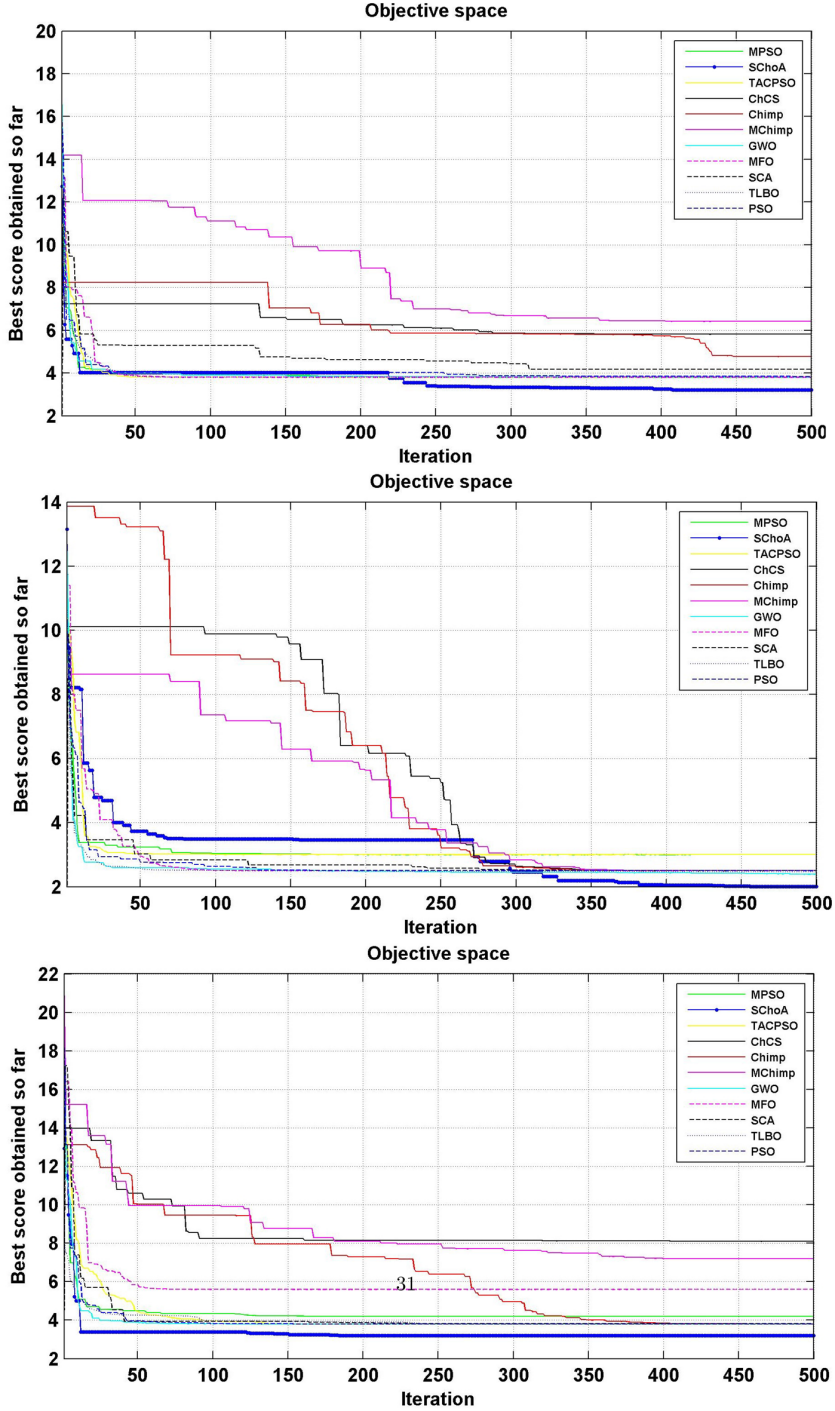
The convergence graph of algorithms on single-objective TDF1IIR, TDF2IIR and DF2IIR digital filters.

**Figure 9:**
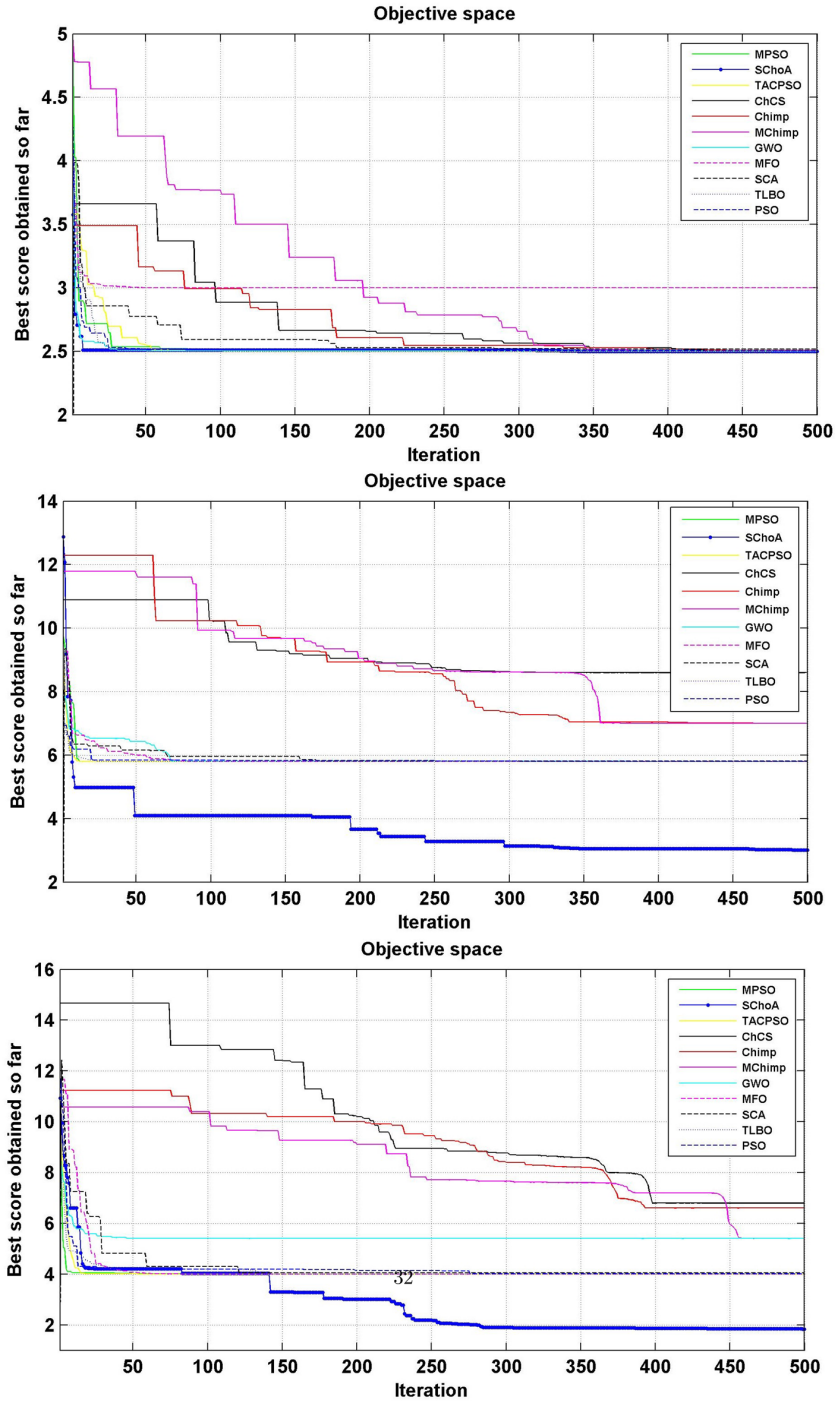
The convergence graph of algorithms on single-objective CASIIR, SLATIIR and DLATIIR digital filters.

**Figure 10:**
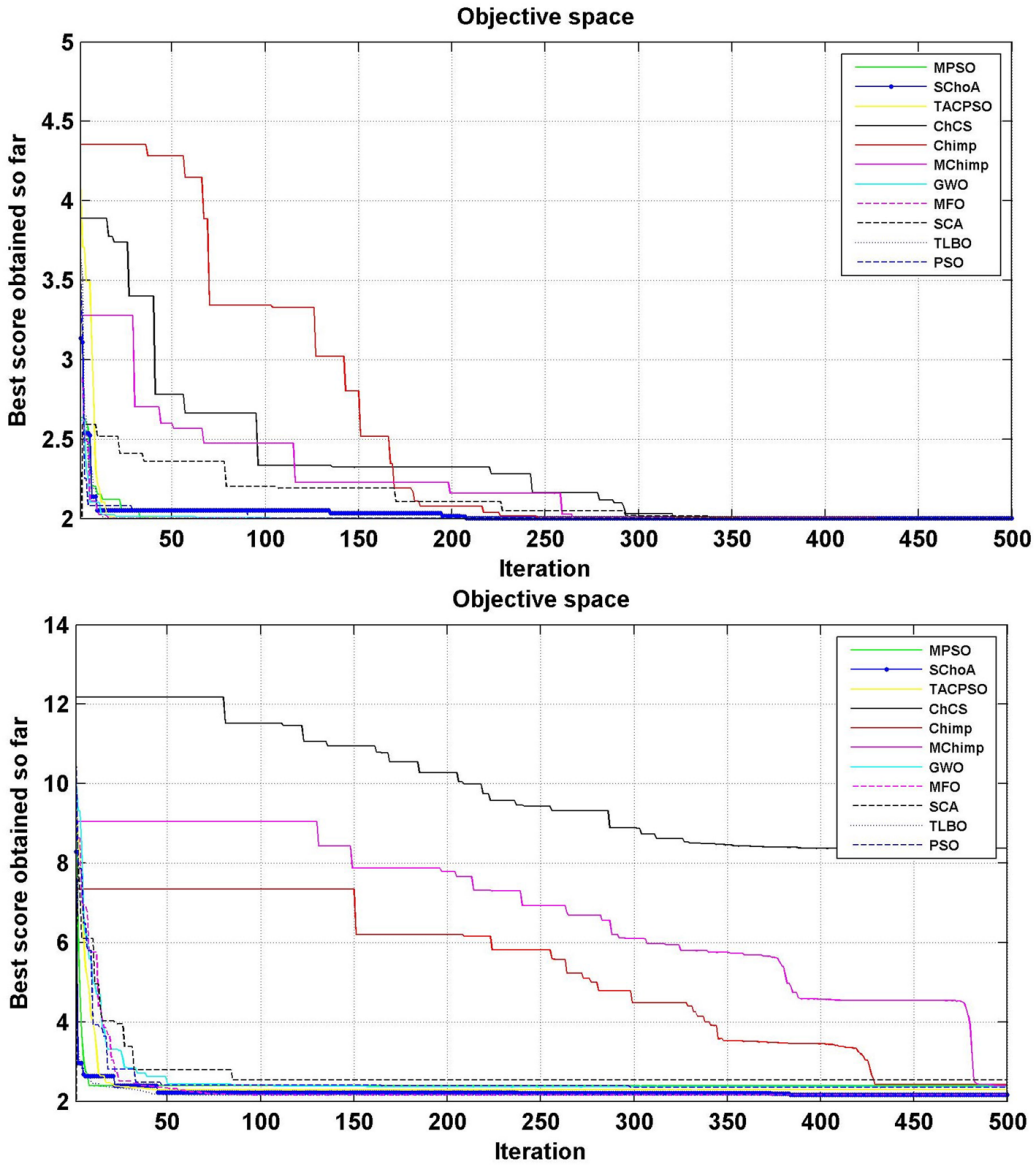
The convergence graph of algorithms on single-objective PARIIR and LADIIR digital filters.

The numerical solutions of the digital filters have been reported in Tables 5, 6, 7, 8, 9, 10, 11, 12, 13, 14, 15, 16, 17, 18, 19, 20, 21, 22, 23, 24, 25, 26, 27, 28, 29, 30, 31, 32, 33 and 34 in the terms of least minimum, highest maximum, average, standard deviation, execution time, occupied area and speed respectively. The performance of the algorithms have been illustrated over single and multi-objective functions. Under this study have been considered two categories of functions for evaluated the high level synthesis of the digital filters such as (1) 14-single objective digital filters and (2) 14-multi-objective digital filters. In Tables 5, 6, 7, 8, 9, 10, 11, 12, 13, 14, 15, 16, 17, 18, have illustrated the performance of the algorithms on the 14-single objective digital filters. The numerical solutions in these tables shows that the proposed method gives the best score as comparison to others. In Figs. 6, 7, 8, 9 and 10 of these filters also proven that the proposed algorithm is able to provide the best optima and accurate solution in the least time and in the least number of iterations or runs. So, the proposed algorithm can be able to prove its own efficiency to reduce the complexity of these filters.

**Table 5:** The global optimal results of the algorithms on single-objective direct form FIR filter.

Algorithm	Best score	Best max	Mean	SD
**SChoA**	**2.001**	**210.9273**	**2.5948**	**1.2636**
MPSO	6.6	17.025	6.6645	2.675
TACPSO	9.2	27.1034	9.4398	1.6964
ChCS	12.866	25.446	16.526	4.5525
Chimp	13.0723	23.3669	16.541	3.9507
MChimp	13.0784	21.7749	16.2532	3.3444
GWO	4.848	20.4637	5.2975	1.6976
MFO	4.8	21.4217	5.3021	2.1577
SCA	5.956	17.3476	6.443	1.4032
TLBO	4.8	17.4918	5.236	1.3733
PSO	4.8132	25.245	5.2916	1.6579

The bold values show the best solutions for problems.

**Table 6:** The global optimal results of the algorithms on single-objective lattice form FIR filter.

Algorithm	Best score	Best max	Mean	SD
**SChoA**	**2**	**8.7279**	**2.0438**	**0.3101**
MPSO	5.0211	12.0759	5.0548	0.3904
TACPSO	5.22	9.5391	50.866	0.4684
ChCS	8.0374	10.087	8.6119	1.1925
Chimp	5.0001	10.9958	8.0069	1.4896
MChimp	5.0001	10.8877	7.0041	1.9999
GWO	5.1021	11.4891	5.0486	0.402
MFO	5.0236	9.1648	5.1644	0.5723
SCA	5.0281	6.6956	5.0664	0.313
TLBO	5.0126	7.2683	5.022	0.4521
PSO	5.2981	9.35	5.0453	0.3978

The bold values show the best solutions for problems.

**Table 7:** The global optimal results of the algorithms on single-objective cascade form FIR filter.

Algorithm	Best score	Best max	Mean	SD
**SChoA**	**2**	**10.5065**	**2.3025**	**0.3447**
MPSO	2.001	9.3818	2.942	0.868
TACPSO	2.9	9.247	3.0367	0.65831
ChCS	3.196	6.8026	3.8655	0.9922
Chimp	3.227	9.5168	4.5794	1.5258
MChimp	3.234	9.6698	4.4008	1.4324
GWO	2.903	8.5665	2.9799	0.9398
MFO	2.9706	8.0757	3.0968	0.532
SCA	3.1388	6.3783	3.3795	0.5173
TLBO	2.9035	7.2913	2.9704	0.3702
PSO	2.9025	6.4123	3.0817	0.7337

The bold values show the best solutions for problems.

**Table 8:** The global optimal results of the algorithms on single-objective parallel form FIR filter.

Algorithm	Best score	Best max	Mean	SD
**SChoA**	**2**	**5.4738**	**2.022**	**0.192**
MPSO	2.002	5.1575	2.026	0.2498
TACPSO	2.001	6.4194	2.0833	0.4673
ChCS	2.0016	6.335	2.5378	1.009
Chimp	2.006	7.8854	2.5811	0.9513
MChimp	2.002	6.4369	2.6	1.1154
GWO	2.0014	6.5121	2.057	0.3269
MFO	2.0251	6.9538	2.6812	0.4188
SCA	2.0366	5.2195	2.1308	0.2818
TLBO	2.121	4.2772	2.0341	0.2066
PSO	2.22	7.5669	2.0868	0.4047

The bold values show the best solutions for problems.

**Table 9:** The global optimal results of the algorithms on single-objective transpose direct form FIR filter.

Algorithm	Best score	Best max	Mean	SD
**SChoA**	**2**	**3.6206**	**2.0119**	**0.028**
MPSO	2.0102	2.4326	2.0075	0.0774
TACPSO	2.011	2.7873	2.0114	0.0577
ChCS	2.0048	3.3692	2.2721	0.4116
Chimp	2.7864	3.8314	2.9155	0.2382
MChimp	2.0024	3.1075	2.2946	0.317
GWO	2.0014	3.2125	2.0192	0.0736
MFO	2.1375	3.8634	2.1857	0.1873
SCA	2.0013	3.7071	2.207	0.2173
TLBO	2.0011	2.6426	2.004	0.0336
PSO	2.0033	3.1933	2.0333	0.0881

The bold values show the best solutions for problems.

**Table 10:** The global optimal results of the algorithms on single-objective direct form IIR filter.

Algorithm	Best score	Best max	Mean	SD
**SChoA**	**3.2**	**17.6002**	**3.9344**	**0.891**
MPSO	11.8	14.5335	11.8185	0.172
TACPSO	5.4613	12.6305	5.6023	0.7837
ChCS	6.0057	13.4063	7.6548	2.4924
Chimp	5.0039	16.2935	7.5746	3.2657
MChimp	5.9984	18.6078	7.6327	2.6068
GWO	3.8025	12.1505	3.9137	0.5437
MFO	4.2	13.7726	4.4028	1.1605
SCA	3.9341	19.219	5.2136	1.9907
TLBO	3.8	14.7553	3.9283	0.7514
PSO	3.8054	17.9583	4.0331	1.4121

The bold values show the best solutions for problems.

**Table 11:** The global optimal results of the algorithms on single-objective transpose direct form IIR-1 filter.

Algorithm	Best score	Best max	Mean	SD
**SChoA**	**3.202**	**12.7328**	**3.6757**	**0.6346**
MPSO	3.8	12.2121	3.9407	0.6259
TACPSO	3.8	11.0269	3.9342	0.7674
ChCS	5.8134	7.2297	6.3365	0.75
Chimp	4.767	8.2288	6.4463	1.2141
MChimp	6.4115	14.1893	8.561	2.3742
GWO	3.8025	17.1272	3.9723	0.8946
MFO	3.8	13.1308	4.0081	0.9875
SCA	4.1692	12.5974	4.7104	0.9022
TLBO	3.8565	11.818	3.9181	0.6547
PSO	3.8348	16.1025	4.0567	0.8181

The bold values show the best solutions for problems.

**Table 12:** The global optimal results of the algorithms on single-objective transpose direct form IIR-2 filter.

Algorithm	Best score	Best max	Mean	SD
**SChoA**	**2.0001**	**12.5078**	**2.9885**	**1.4321**
MPSO	3	12.4903	3.1886	0.883
TACPSO	2.4995	11.521	2.6825	0.9083
ChCS	4	7.9303	5.7231	1.7345
Chimp	4.0007	11.673	6.9392	2.5919
MChimp	2.5025	13.7468	6.1608	3.548
GWO	2.4954	10.0096	2.6144	0.5615
MFO	2.794	12.999	3.1936	1.532
SCA	2.5071	11.1921	2.7635	0.7914
TLBO	2.4188	6.3385	2.4787	0.3085
PSO	2.4933	9.9725	2.6297	0.5863

The bold values show the best solutions for problems.

**Table 13:** The global optimal results of the algorithms on single-objective direct form IIR-2 filter.

Algorithm	Best score	Best max	Mean	SD
**SChoA**	**3.2**	**12.9193**	**3.3646**	**0.8452**
MPSO	4.2	17.3297	4.3595	0.9658
TACPSO	3.8	13.9067	4.1401	1.18
ChCS	8.0985	13.968	8.7321	1.4725
Chimp	3.8059	13.111	6.6859	2.7878
MChimp	7.2021	21.7115	8.5815	1.9148
GWO	3.8081	18.757	3.9466	0.9807
MFO	5.6	16.4038	0.8728	1.2778
SCA	3.8125	17.2306	4.0884	1.2642
TLBO	3.8253	8.1135	3.976	1.4236
PSO	3.8555	18.922	3.989	1.0307

The bold values show the best solutions for problems.

**Table 14:** The global optimal results of the algorithms on single-objective cascade form IIR-2 filter.

Algorithm	Best score	Best max	Mean	SD
**SChoA**	**2.5**	**3.575**	**2.5108**	**0.0531**
MPSO	2.5005	4.9231	2.5279	0.1585
TACPSO	2.5216	3.5783	2.5382	0.1522
ChCS	2.5058	3.6604	2.7769	0.3768
Chimp	2.55	3.4886	2.7318	0.3048
MChimp	2.5038	4.9792	3.0672	0.6584
GWO	2.5004	3.936	2.5105	0.0783
MFO	3.007	3.663	3.0072	0.0549
SCA	2.5152	3.985	2.581	0.1983
TLBO	2.5164	4.0363	2.5219	0.133
PSO	2.5023	4.3532	2.5245	0.1113

The bold values show the best solutions for problems.

**Table 15:** The global optimal results of the algorithms on single-objective SS lattice form IIR filter.

Algorithm	Best score	Best max	Mean	SD
**SChoA**	**3.008**	**12.8731**	**5.6723**	**0.9228**
MPSO	5.8	9.7896	5.8452	0.3542
TACPSO	5.8	8.8013	5.8184	0.8814
ChCS	8.6	10.888	9.2427	0.8772
Chimp	7.0058	12.2945	8.6923	1.7725
MChimp	7.001	11.7942	8.92525	1.6074
GWO	5.8002	7.8945	5.9101	0.8274
MFO	5.8003	9.2786	5.8841	0.6319
SCA	5.8134	9.3424	5.9071	0.8333
TLBO	5.821	7.0899	5.8131	0.9801
PSO	5.8006	6.9432	5.8376	0.7524

The bold values show the best solutions for problems.

**Table 16:** The global optimal results of the algorithms on single-objective DS-lattice form IIR filter.

Algorithm	Best score	Best max	Mean	SD
**SChoA**	**2**	**10.9322**	**2.8544**	**1.2677**
MPSO	4	10.285	4.2051	1.402
TACPSO	4.001	10.048	4.0543	0.4422
ChCS	6.8	14.6637	10.0989	2.7477
Chimp	6.60003	11.2271	9.0192	1.6648
MChimp	5.401	10.575	8.3921	1.5678
GWO	5.3987	9.0144	5.4457	0.5861
MFO	4.0308	11.6615	4.2051	1.002
SCA	4.0549	12.4323	4.3074	0.8712
TLBO	4	8.1319	4.1035	0.6592
PSO	4.0098	11.8028	4.1792	0.6182

The bold values show the best solutions for problems.

**Table 17:** The global optimal results of the algorithms on single-objective parallel form IIR-2 filter.

Algorithm	Best score	Best max	Mean	SD
**SChoA**	**2**	**4.2505**	**2.0195**	**0.1317**
MPSO	2.0071	5.4267	2.0371	0.156
TACPSO	2.02	5.5167	2.0503	0.23
ChCS	2.0001	3.7446	2.035	0.203
Chimp	2.005	4.1569	2.5059	0.3589
MChimp	2.0074	4.3069	2.0832	0.4109
GWO	2.005	4.6426	2.0102	0.9913
MFO	2.115	4.5478	2.0275	0.6174
SCA	2.0119	3.9378	2.0593	0.9146
TLBO	2.0151	4.4391	2.0126	0.1107
PSO	2.0002	4.5202	2.0213	0.1946

The bold values show the best solutions for problems.

**Table 18:** The global optimal results of the algorithms on single-objective lattice ladder form IIR filter.

Algorithm	Best score	Best max	Mean	SD
**SChoA**	**2.1665**	**8.2876**	**2.2429**	**0.291**
MPSO	2.3917	9.2255	2.4234	0.3751
TACPSO	2.2917	9.5753	2.3845	0.6318
ChCS	8.3662	12.1782	9.8557	1.4494
Chimp	2.4272	7.3386	5.2377	1.8086
MChimp	2.3932	9.0506	6.8039	1.8742
GWO	2.3591	10.2187	2.5273	0.7707
MFO	2.2583	8.6259	2.322	0.7706
SCA	2.5332	8.0528	2.6882	0.6164
TLBO	2.2583	4.2576	2.1846	0.3147
PSO	2.3472	10.7821	2.5034	0.9692

The bold values show the best solutions for problems.

**Table 19:** The execution time of the algorithms on single-objective digital filters.

Digital filters	SChoA	MPSO	TAPSO	ChCS	Chimp	MChimp	GWO	MFO	SCA	TLBO	PSO
DF-FIR	**1.57**	2.22	1.93	11.74	3.711	3.886	3.71	6.015	1.77	5.8	2.06
LATFIR	**0.783**	1.305	1.075	5.926	2.016	2.866	**2.15**	3.426	0.94	3.2	1.17
CASFIR	**0.913**	1.533	1.257	7.306	2.42	3.661	**2.59**	4.223	1.1	4	1.37
PARFIR	**0.993**	1.558	1.306	7.397	2.45	3.51	2.6	4.09	1.16	3.9	1.42
TDFFIR	**0.568**	1.04	0.836	4.42	1.57	2.2	1.69	2.71	0.71	2.5	0.92
DF1IIR	**1.034**	1.805	1.44	7.965	2.77	3.923	2.98	4.64	1.25	4.4	1.6
TDF1IIR	**1.02**	1.794	1.443	8.013	2.751	3.914	2.95	4.64	1.24	4.4	1.59
TDF2IIR	**1.064**	1.92	1.52	8.31	2.89	4.106	3.12	4.92	1.3	4.6	1.71
DF2IIR	**1.019**	1.798	1.43	7.95	2.73	3.87	2.93	4.61	1.24	4.1	1.59
CASIIR	**0.557**	1.04	0.826	4.43	1.55	2.216	1.69	2.73	0.7	2.5	0.91
SLATIIR	**0.68**	1.31	1.03	5.51	1.95	2.75	2.11	3.38	0.87	3.2	1.15
DLATIIR	**1.05**	1.95	1.54	8.33	2.94	4.165	3.17	4.98	1.3	4.7	1.73
PARIIR	**0.777**	1.336	1.08	6.03	2.064	2.932	2.21	3.5	0.94	3.3	1.19
LADIIR	**1.675**	2.33	2.03	12.42	3.9	5.63	4.08	6.328	1.87	6.1	2.17

The bold values show the best solutions for problems.

**Figure 11:**
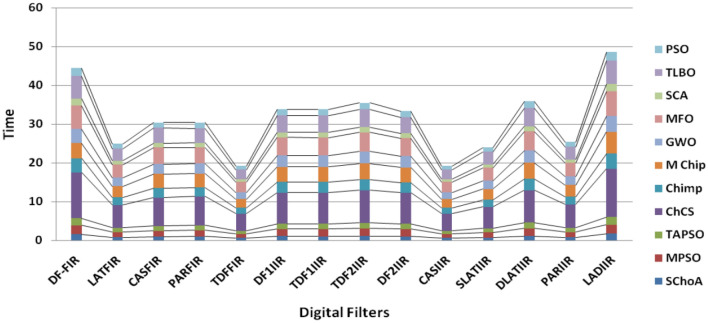
The graph of execution time of algorithms on single-objective digit filters.

**Figure 12:**
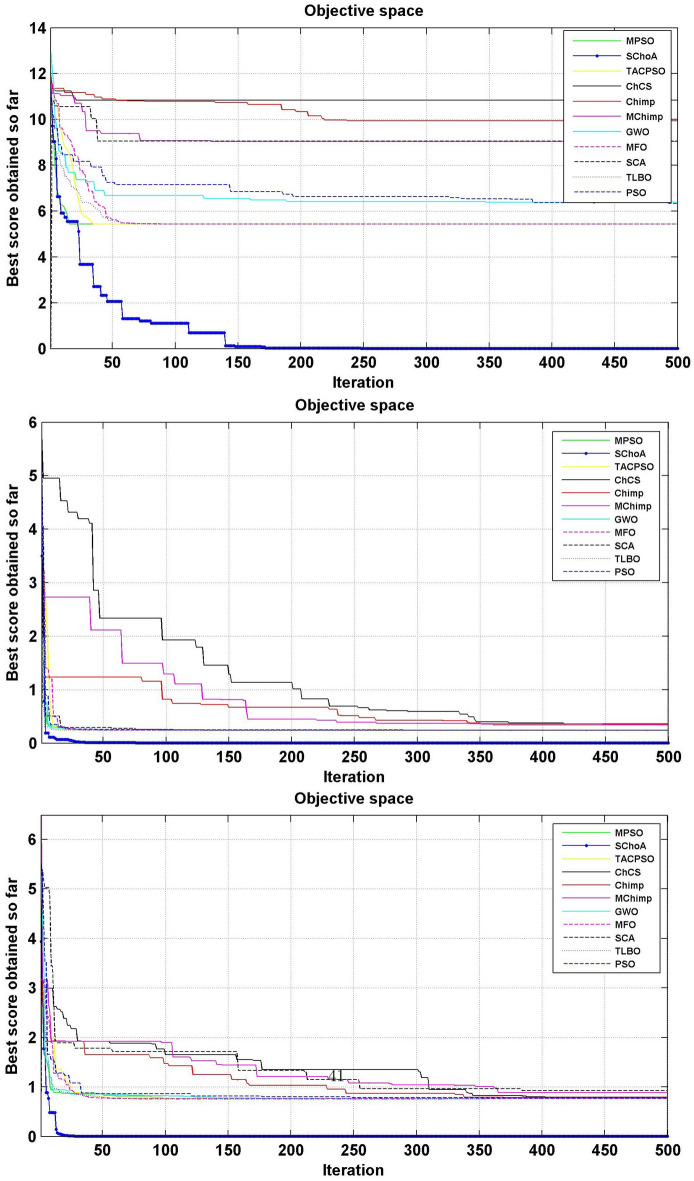
The convergence graph of algorithms on multi-objective DF-FIR, LATFIR and CASFIR digital filters.

**Figure 13:**
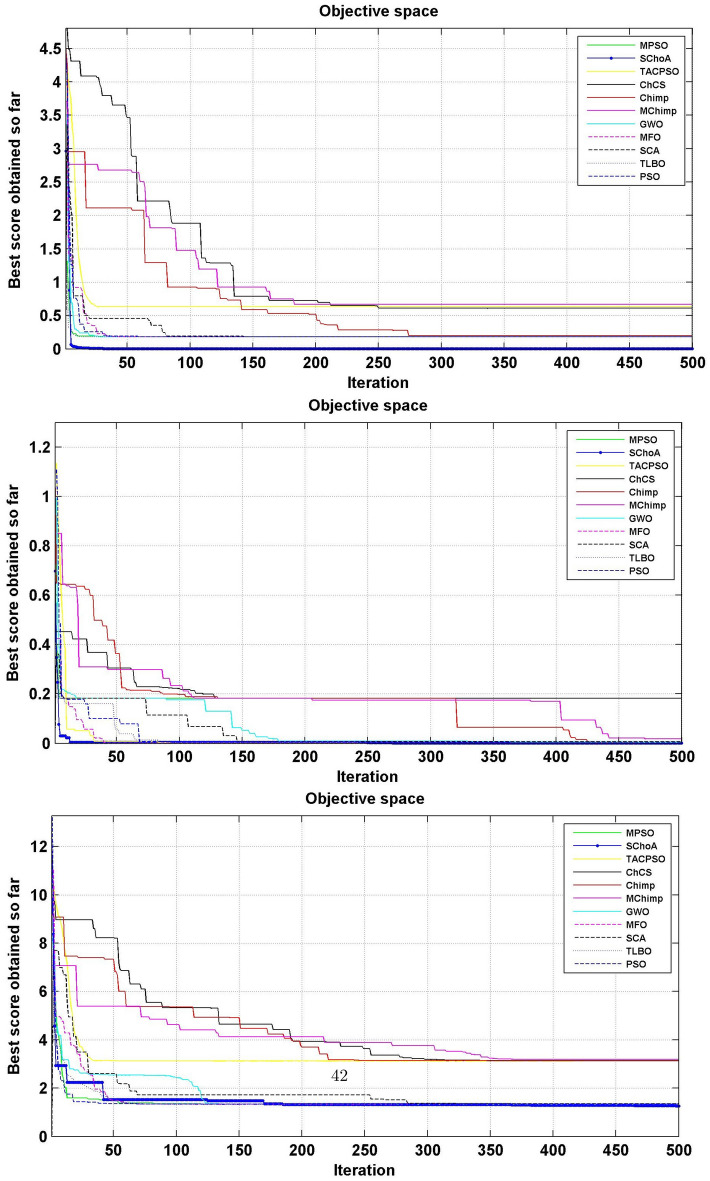
The convergence graph of algorithms on multi-objective PARFIR, TDFFIR and DF1IIR digital filters.

**Figure 14:**
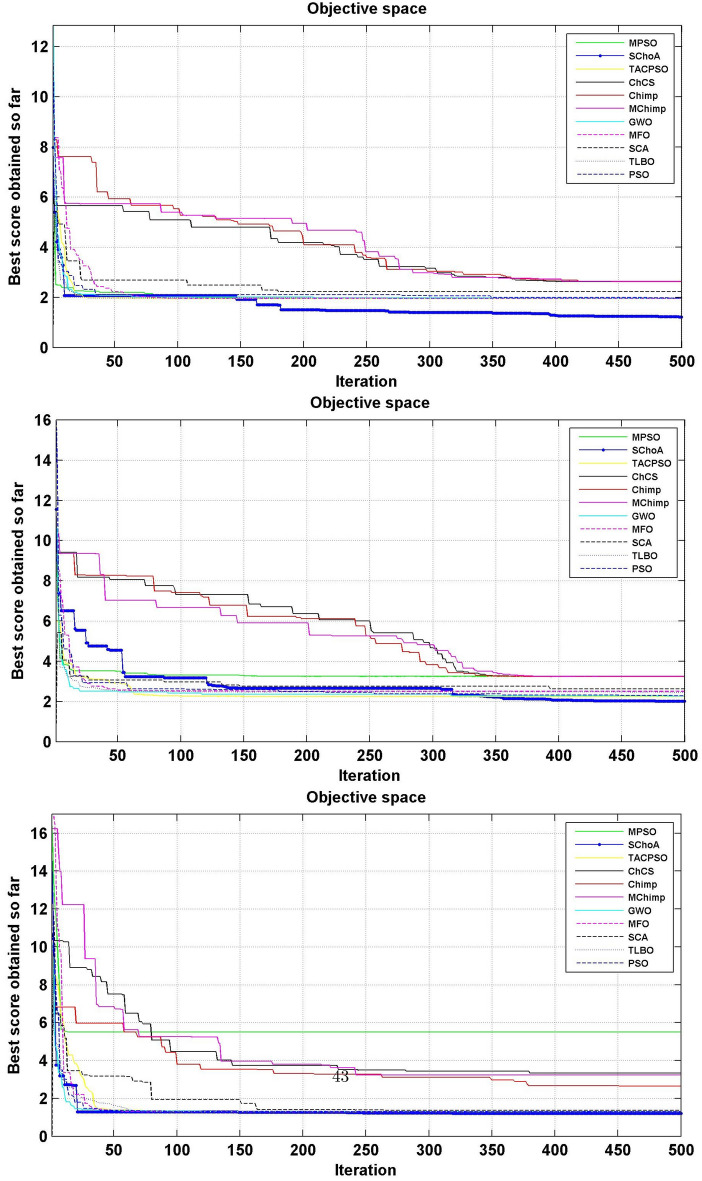
The convergence graph of algorithms on multi-objective TDF1IIR, TDF2IIR and DF2IIR digital filters.

**Figure 15:**
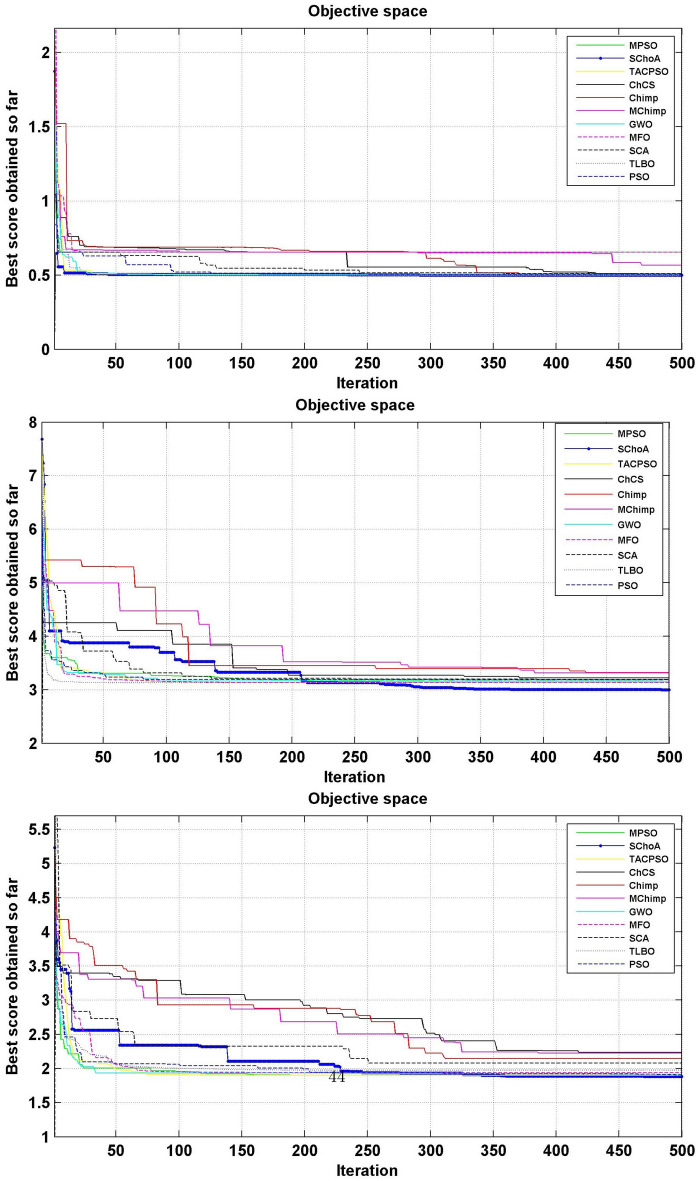
The convergence graph of algorithms on multi-objective CASIIR, SLATIIR and DLATIIR digital filters.

**Figure 16:**
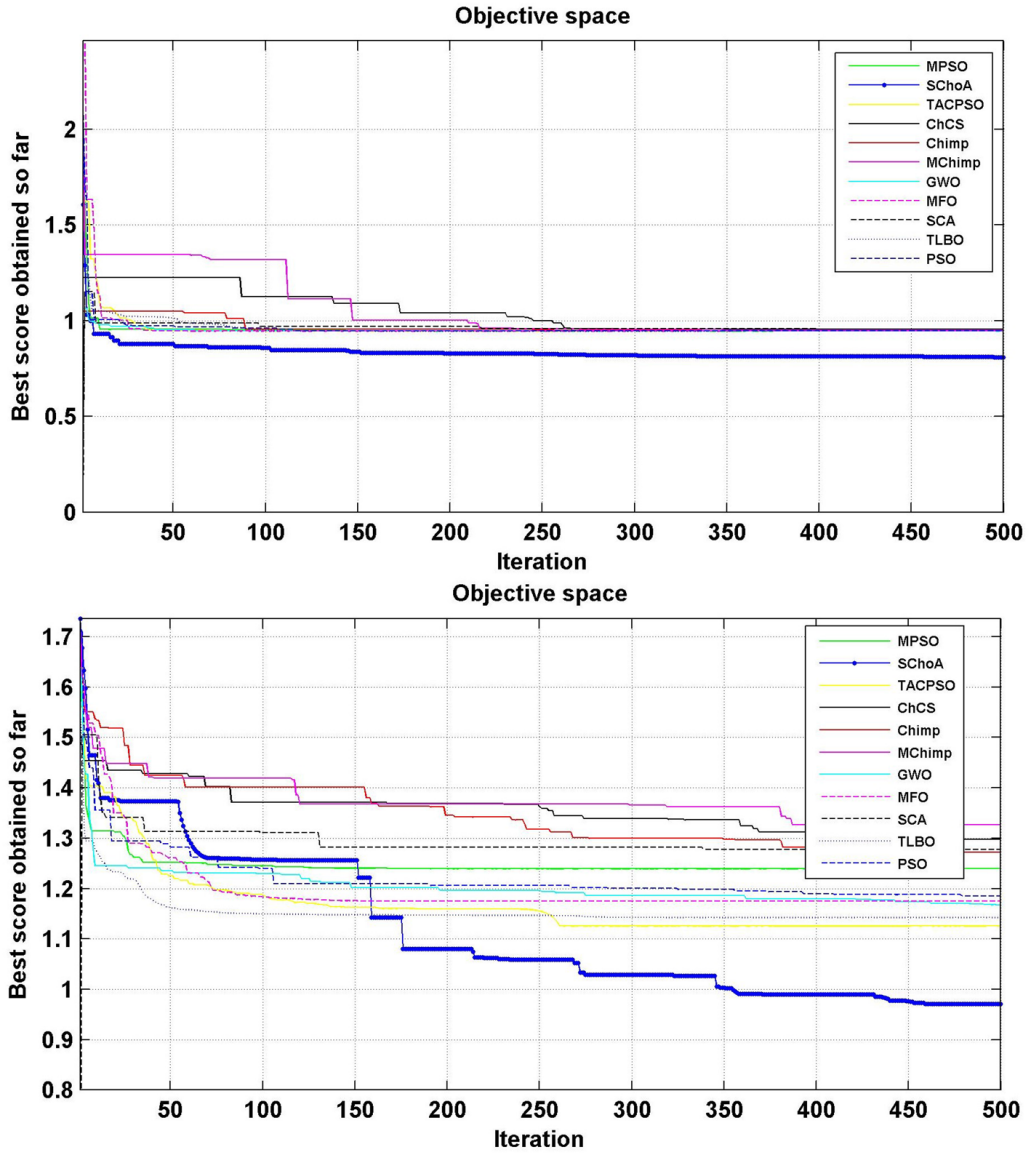
The convergence graph of algorithms on multi-objective PARIIR and LADIIR digital filters.

Similarly, in Tables 20, 21, 22, 23, 24, 25, 26, 27, 28, 29, 30, 31, 32, 33, have been reported the algorithms solutions on the multi-objective digital filters. The outputs of the algorithms are revealed that the proposed method is capable of presenting the best and accurate global optima solutions on these multi-objective functions. The convergence performance of the algorithms have been plotted through the Figs. 12, 13, 14, 15 and 16. These graphs give the proof of the best solutions trapping performance of the algorithms. And proven that the proposed method easily and quickly trapping the best and accurate global solution with the least number of iterations and time.

**Table 20:** The global optimal results of the algorithms on multi-objective of direct form FIR filter.

Algorithm	Best score	Best max	Mean	SD
SChoA	**0.0046**	**11.8261**	**0.6708**	**1.054**
MPSO	5.4383	11.6974	5.4888	0.3805
TACPSO	5.4383	11.4401	5.6124	0.829
ChCS	10.8411	11.358	10.856	0.0759
Chimp	9.943	11.9379	10.3059	0.4513
MChimp	9.00396	11.5773	9.1815	0.4439
GWO	6.3861	12.9561	6.5973	0.604
MFO	5.4383	17.5802	5.6966	0.9476
SCA	9.049	10.8017	9.1355	0.5581
TLBO	5.4383	10.7239	5.5887	0.5822
PSO	6.3402	10.9351	6.8387	0.6043

The bold values show the best solutions for problems.

**Table 21:** The global optimal results of the algorithms on multi-objective lattice form FIR filter.

Algorithm	Best score	Best max	Mean	SD
**SChoA**	**0.001098**	**4.005**	**0.121**	**0.195**
MPSO	0.24138	3.0009	0.2556	0.161
TACPSO	0.2385	3.4205	0.2724	0.2842
ChCS	0.36038	5.7828	1.2438	1.1965
Chimp	0.33717	1.2334	0.6302	0.3216
MChimp	0.35147	2.7265	0.8032	0.7314
GWO	0.24181	1.971	0.2554	0.1235
MFO	0.24138	3.2128	0.2755	0.2571
SCA	0.24312	4.0598	0.2655	0.1923
TLBO	0.239	1.4698	0.245	0.0688
PSO	0.24192	5.4632	0.2693	0.2744

The bold values show the best solutions for problems.

**Table 22:** The global optimal results of the algorithms on multi-objective cascade form FIR filter.

Algorithm	Best score	Best max	Mean	SD
**SChoA**	**0.000329**	**5.9363**	**0.0329**	**0.2621**
MPSO	0.76	4.4759	0.7954	0.2038
TACPSO	0.76	3.2125	0.8192	0.2746
ChCS	0.7938	4.12	1.3391	0.5144
Chimp	0.7818	6.0987	1.1026	0.4374
MChimp	0.88428	6.481	1.2928	0.4905
GWO	0.76723	5.4576	0.8195	0.3095
MFO	0.7673	3.1406	0.8107	0.2705
SCA	0.9269	5.869	1.3091	0.5904
TLBO	0.7659	4.4726	0.7949	0.241
PSO	0.7733	5.5713	0.8038	0.388

The bold values show the best solutions for problems.

**Table 23:** The global optimal results of the algorithms on multi-objective parallel form FIR filter.

Algorithm	Best score	Best max	Mean	SD
**SChoA**	**0.0003139**	**4.2657**	**0.0202**	**0.2194**
MPSO	0.18182	1.312	0.1894	0.0788
TACPSO	0.63077	4.2677	0.6911	0.3971
ChCS	0.60953	4.8039	1.1896	1.0714
Chimp	0.19982	4.5335	0.6468	0.7323
MChimp	0.6607	3.7075	1.0543	0.706
GWO	0.18182	3.5682	0.2035	0.2158
MFO	0.1869	3.1628	0.2208	0.2365
SCA	0.18138	3.3655	0.2491	0.2542
TLBO	0.18163	1.8273	0.1877	0.0807
PSO	0.18138	4.4616	0.2271	0.3086

The bold values show the best solutions for problems.

**Table 24:** The global optimal results of the algorithms on multi-objective transpose direct form FIR filter.

Algorithm	Best score	Best max	Mean	SD
**SChoA**	**1.40E−12**	**1.6981**	**0.00152**	**0.0367**
MPSO	0.1818	0.8401	0.1839	0.032
TACPSO	1.70E−03	1.1337	0.0163	0.1056
ChCS	0.1813	0.4521	0.211	0.0672
Chimp	0.0026	1.0916	0.1772	0.1588
MChimp	0.0175	0.8503	0.19	0.1289
GWO	0.0045	0.9938	0.061	0.0988
MFO	2.69E−04	0.4235	0.0109	0.0502
SCA	6.33E−03	0.3667	0.044	0.0687
TLBO	6.24E−03	0.4219	0.0187	0.0539
PSO	1.90E−03	1.1095	0.0265	0.0955

The bold values show the best solutions for problems.

**Table 25:** The global optimal results of the algorithms on multi-objective direct form of IIR filter.

Algorithm	Best score	Best max	Mean	SD
**SChoA**	**1.2575**	**10.8814**	**1.4798**	**0.59**
MPSO	1.3364	8.7989	1.4343	0.6093
TACPSO	3.1224	10.8766	3.3147	0.9774
ChCS	3.1423	11.8204	4.4183	1.7818
Chimp	3.128	9.2131	4.1575	1.4901
MChimp	3.19	7.0705	4.0423	0.9261
GWO	1.3364	8.3395	1.6751	0.7541
MFO	1.352	11.063	1.5481	0.8915
SCA	1.3579	7.7763	1.8011	0.9896
TLBO	1.3345	7.8291	1.4539	0.4921
PSO	1.3356	11.288	1.4148	0.6667

The bold values show the best solutions for problems.

**Table 26:** The global optimal results of the algorithms on multi-objective transpose direct form IIR-1 filter.

Algorithm	Best score	Best max	Mean	SD
**SChoA**	**1.2212**	**11.9833**	**1.6533**	**0.5468**
MPSO	1.9538	6.2299	2.0264	0.276
TACPSO	1.9557	6.7376	2.0407	0.506
ChCS	2.6172	6.0942	3.8342	1.1254
Chimp	2.6271	8.2912	4.113	1.5034
MChimp	2.6467	7.5037	4.0924	1.3276
GWO	1.9607	12.883	2.0724	0.6413
MFO	1.9585	8.3622	2.1725	0.8652
SCA	2.2205	5.4305	2.4115	0.4854
TLBO	1.9601	7.3456	2.0096	0.3732
PSO	1.9612	11.7147	2.1502	0.6205

The bold values show the best solutions for problems.

**Table 27:** The global optimal results of the algorithms on multi-objective transpose direct form IIR-2 filter.

Algorithm	Best score	Best max	Mean	SD
**SChoA**	**2.0165**	**16.0073**	**2.8357**	**1.0838**
MPSO	3.2512	10.3841	3.3284	0.4046
TACPSO	2.2513	5.9836	2.3972	0.4758
ChCS	3.2525	9.4866	5.528	1.9865
Chimp	3.2352	9.3676	5.3458	1.9906
MChimp	3.2515	10.3747	5.2124	1.7995
GWO	2.2642	10.6177	2.4176	0.5913
MFO	2.5655	8.5887	2.643	0.6935
SCA	2.6265	5.4223	2.8308	0.3355
TLBO	2.4422	11.9996	2.5455	0.5322
PSO	2.2824	11.553	2.5888	0.8969

The bold values show the best solutions for problems.

**Table 28:** The global optimal results of the algorithms on multi-objective direct form IIR-2 filter.

Algorithm	Best score	Best max	Mean	SD
**SChoA**	**1.2025**	**16.372**	**1.3595**	**0.8439**
MPSO	5.5071	16.367	5.5909	0.7689
TACPSO	1.2976	9.3635	1.536	1.0161
ChCS	3.3397	10.7041	4.3451	1.769
Chimp	2.6432	6.8168	3.6225	1.1516
MChimp	3.236	16.237	4.4667	2.4085
GWO	1.2976	10.2413	1.3891	0.6186
MFO	1.2979	16.9928	1.5535	1.57
SCA	1.3658	11.6727	1.8209	1.012
TLBO	1.2976	8.3309	1.4338	0.5666
PSO	1.2971	14.609	1.4434	0.9212

The bold values show the best solutions for problems.

**Table 29:** The global optimal results of the algorithms on multi-objective cascade form IIR-2 filter.

Algorithm	Best score	Best max	Mean	SD
**SChoA**	**0.5**	**2.013**	**0.5079**	**0.0741**
MPSO	0.65385	1.9582	0.6575	0.0601
TACPSO	0.5012	1.728	0.5128	0.0975
ChCS	0.50578	1.0401	0.6041	0.0847
Chimp	0.50526	2.1273	0.6325	0.1598
MChimp	0.5671	1.0451	0.6503	0.0487
GWO	0.501	1.8308	0.5132	0.0791
MFO	0.6538	2.164	0.6671	0.107
SCA	0.5091	0.6598	0.5288	0.0486
TLBO	0.5611	1.1453	0.5077	0.0509
PSO	0.50368	0.8542	0.5288	0.0486

The bold values show the best solutions for problems.

**Table 30:** The global optimal results of the algorithms on multi-objective SS lattice form IIR filter.

Algorithm	Best score	Best max	Mean	SD
**SChoA**	**3.0008**	**7.6799**	**3.317**	**0.4696**
MPSO	3.1822	4.9868	3.2335	0.1301
TACPSO	3.1337	7.5895	3.2178	0.3994
ChCS	3.216	4.5375	3.5132	0.391
Chimp	3.317	5.4233	3.7791	0.73
MChimp	3.314	6.3322	3.8044	0.5872
GWO	3.15	6.1241	3.2197	0.2744
MFO	3.124	5.3379	3.1013	0.2628
SCA	3.192	5.1858	3.3309	0.3969
TLBO	3.13	6.7998	3.15	0.2338
PSO	3.185	6.8619	3.2273	0.1997

The bold values show the best solutions for problems.

**Table 31:** The global optimal results of the algorithms on multi-objective DS-lattice form IIR filter.

Algorithm	Best score	Best max	Mean	SD
**SChoA**	**1.803**	**5.2311**	**2.1122**	**0.344**
MPSO	1.899	3.7215	1.9432	0.1542
TACPSO	1.893	4.373	1.9619	0.2968
ChCS	2.23	3.9487	2.7475	0.4281
Chimp	2.14	5.21	2.6807	0.557
MChimp	2.22	4.8219	2.8517	0.437
GWO	1.91	4.458	1.955	0.14448
MFO	1.93	3.356	2.007	0.2276
SCA	2.07	5.099	2.2719	0.3788
TLBO	1.976	3.646	2.0231	0.159
PSO	1.9072	4.5145	2.0091	0.2161

The bold values show the best solutions for problems.

**Table 32:** The global optimal results of the algorithms on multi-objective parallel form IIR-2 filter.

Algorithm	Best score	Best max	Mean	SD
**SChoA**	**0.81**	**2.1299**	**0.9591**	**0.0716**
MPSO	0.946	1.9723	0.93	0.0573
TACPSO	0.945	1.6318	0.9593	0.0711
ChCS	0.954	1.2252	1.0415	0.1016
Chimp	0.947	1.0481	0.9675	0.0351
MChimp	0.953	2.0413	1.0416	0.1606
GWO	0.944	1.0653	0.9502	0.0118
MFO	0.9434	2.4627	0.9664	0.1171
SCA	0.955	1.1534	0.9624	0.0491
TLBO	0.963	1.34267	0.9598	0.0394
PSO	0.949	1.6057	0.8371	0.05

The bold values show the best solutions for problems.

**Table 33:** The global optimal results of the algorithms on multi-objective lattice ladder form IIR filter.

Algorithm	Best score	Best max	Mean	SD
**SChoA**	**0.97**	**1.7351**	**1.1153**	**0.1459**
MPSO	1.2395	1.6089	1.247	0.0302
TACPSO	1.125	1.6106	1.167	0.0703
ChCS	1.295	1.4926	1.3552	0.0424
Chimp	1.271	1.6353	1.3436	0.069
MChimp	1.326	1.721	1.3737	0.0442
GWO	1.67	1.6461	1.2025	0.0397
MFO	1.175	1.5507	1.1931	0.0665
SCA	1.277	1.5052	1.2906	0.0672
TLBO	1.142	1.5474	1.1547	0.035
PSO	1.1845	1.7192	1.219	0.0543

The bold values show the best solutions for problems.

**Figure 17:**
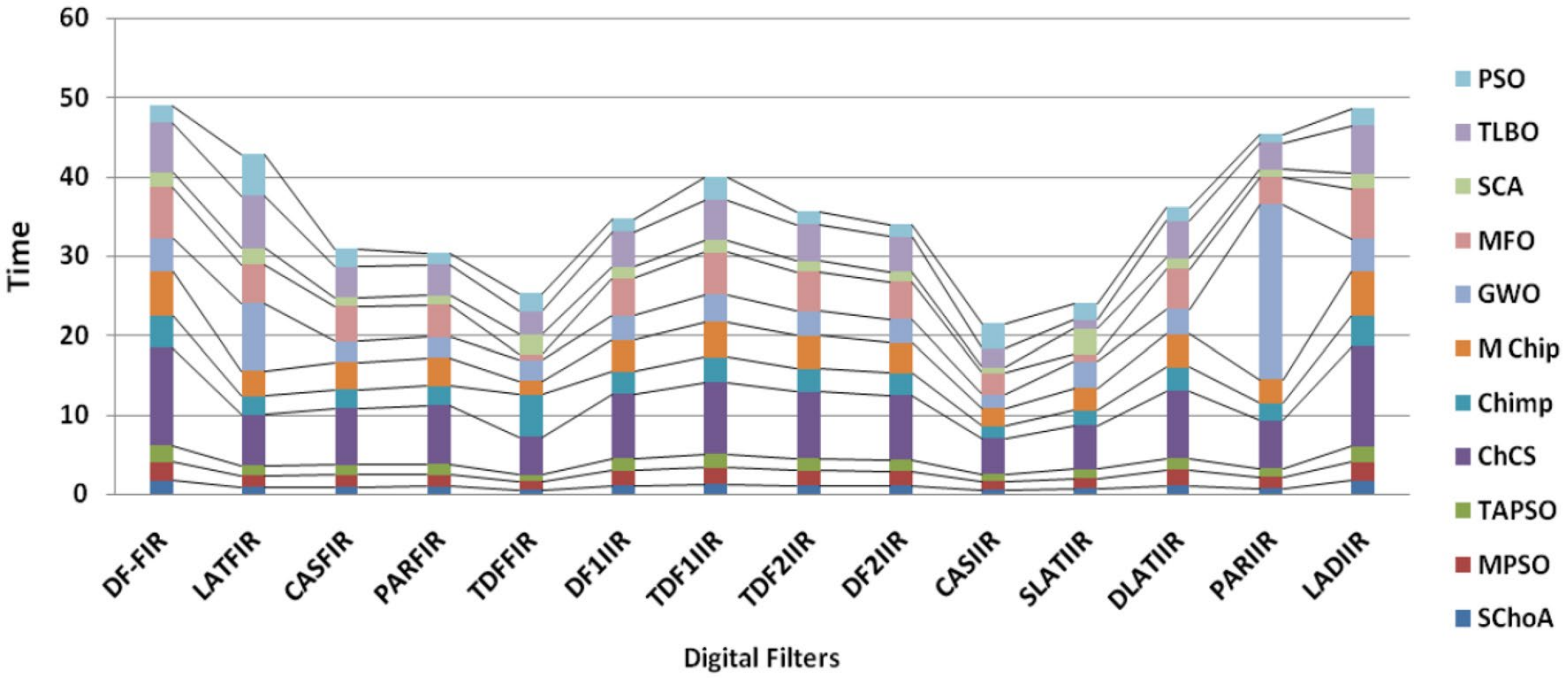
The graph of execution time of algorithms on multi-objective digit filters.

In Tables 19, 20, 21, 22, 23, 24, 25, 26, 27, 28, 29, 30, 31, 32, 33, 34, the execution time of the algorithms on the single and multi-objective digital filters have been illustrated. The results show that the proposed algorithm can be trapping the best global optima solution in the complex search domain easily and fastly outperforms others. The execution time performance of the algorithms on the single and multi-objective digital filters have been plotted through Figs. 11, 12, 13, 14, 15, 16 and 17. These graphs give strong evidence that the proposed method is able to trap the best goal fastly as comparison than others.

**Table 34:** The Execution time of the algorithms on multi-objective digital filters.

Digital filters	SChoA	MPSO	TAPSO	ChCS	Chimp	MChimp	GWO	MFO	SCA	TLBO	PSO
DF-FIR	**1**.**636**	2.43	2.06	12.3	3.96	5.63	4.17	6.49	1.86	6.22	2.23
LATFIR	**0**.**818**	1.51	1.2	6.48	2.28	3.23	8.46	4.88	2.01	6.69	5.34
CASFIR	**0**.**9**	1.53	1.24	7.13	2.35	3.45	2.59	4.41	1.08	3.94	2.35
PARFIR	**0**.**98**	1.44	1.29	7.43	2.45	3.5	2.61	4.07	1.15	3.91	1.55
TDFFIR	**0**.**553**	1.023	0.814	4.83	5.32	1.66	2.67	0.69	2.54	2.9	2.36
DF1IIR	**1**.**085**	1.86	1.511	8.15	2.82	3.99	3.03	4.75	1.31	4.51	1.67
TDF1IIR	**1**.**19**	2.11	1.74	9.02	3.17	4.42	3.42	5.34	1.52	5.05	2.91
TDF2IIR	**1**.**05**	1.93	1.52	8.36	2.93	4.11	3.13	4.93	1.3	4.7	1.71
DF2IIR	**1**.**041**	1.81	1.46	8.1	2.79	3.79	2.98	4.7	1.26	4.45	1.62
CASIIR	**0**.**568**	1.065	0.846	4.48	1.59	2.23	1.72	2.72	0.71	2.36	3.21
SLATIIR	**0**.**703**	1.31	1.045	5.53	1.96	2.76	3.38	0.84	3.21	1.16	2.12
DLATIIR	**1**.**05**	1.95	1.53	8.42	2.94	4.2	3.18	5.02	1.3	4.78	1.72
PARIIR	**0**.**777**	1.34	1.085	6.04	2.22	2.93	22.04	3.52	0.94	3.35	1.19
LADIIR	**1**.**673**	2.33	2.03	12.5	3.91	5.64	4.08	6.29	1.86	6.11	2.17

The bold values show the best solutions for problems.

**Figure 18:**
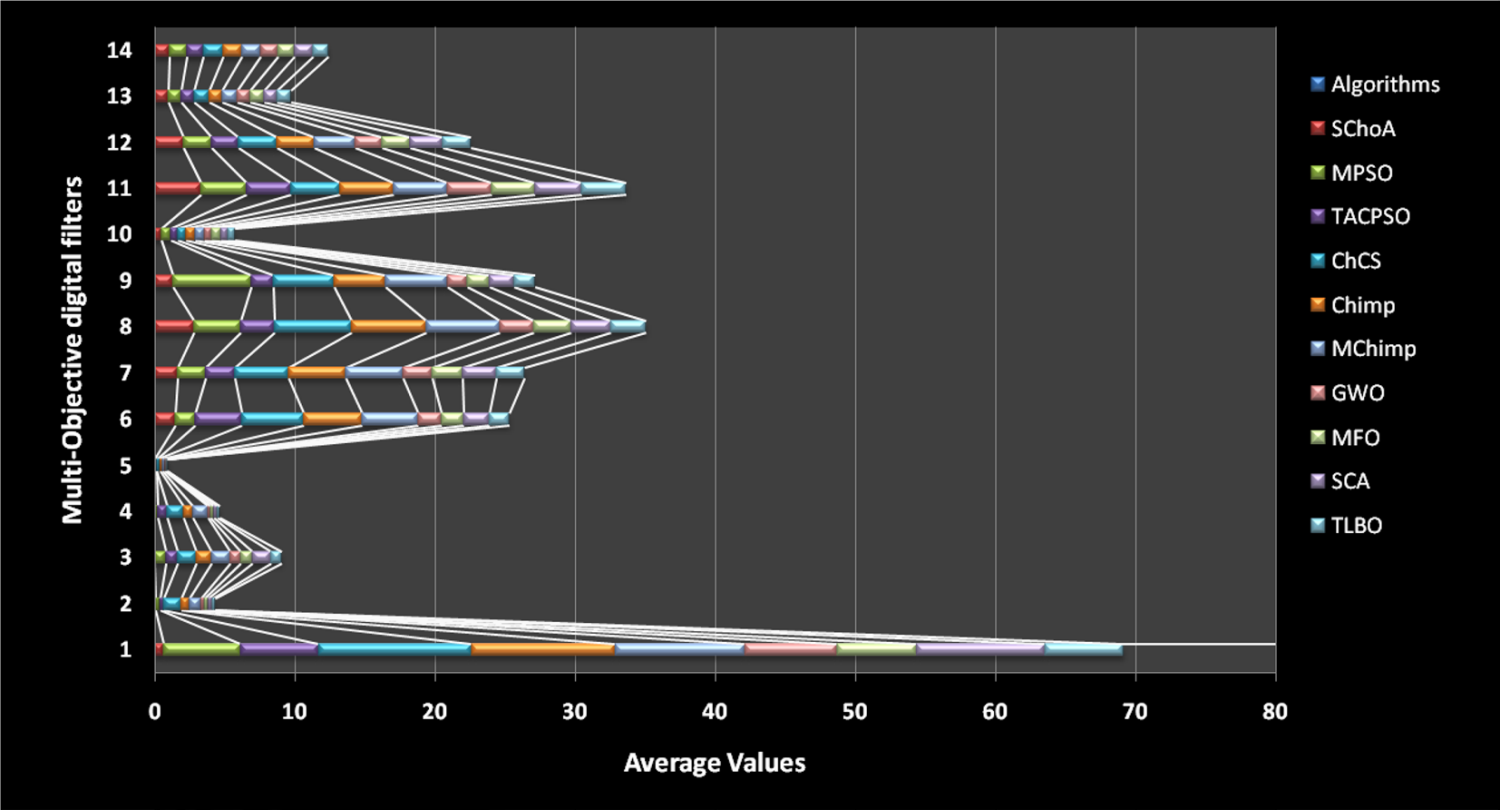
The average values of the proposed algorithm on 14-multi-objective digital filters.

The average values of the algorithms have been illustrated through a Fig. 18. This graph has been plotted over the average values of the algorithms on the multi-objective digital filters, these values are shown in Tables 20, 21, 22, 23, 24, 25, 26, 27, 28, 29, 30, 31, 32, 33. Generally, the least value of mean represents the accuracy of the optimizer algorithm for the best global optima. These figures give strong evidence of the superior accuracy of the proposed algorithm as comparison with others on these multi-objective digital filters. Finally, we can say that the proposed methodology is able to present accurate and superior global optima solutions for these complex filters.

**Figure 19:**
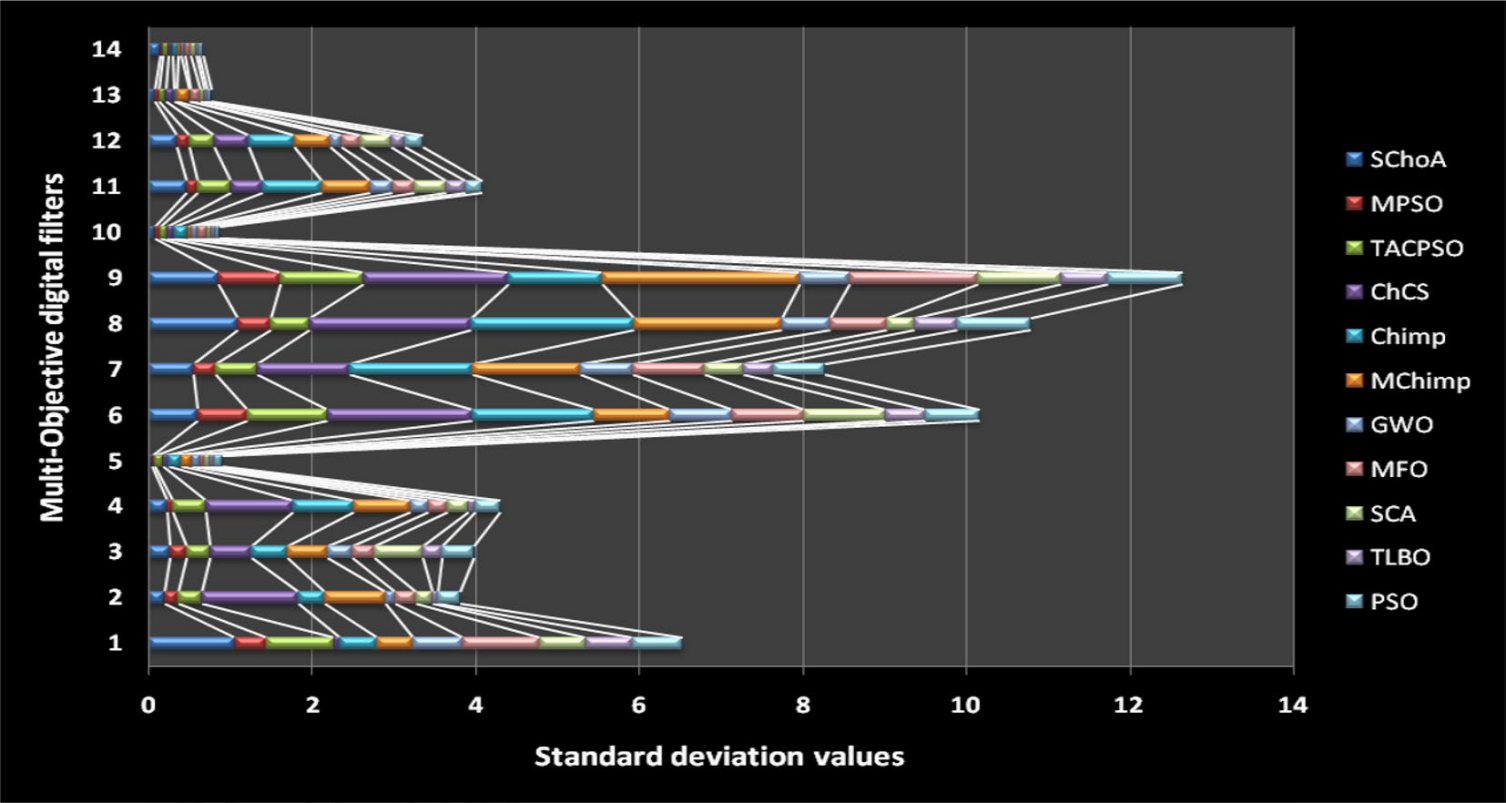
The standard deviation values of the proposed algorithm on 14-multi-objective digital filters.

In this phase, we are discussing the standard deviation values (*sd*) of the algorithms. The *sd* values of each algorithm have been plotted by the graph or Fig. 19 with respect to x-axis and y-axis respectively. These values are illustrates through Tables 20, 21, 22, 23, 24, 25, 26, 27, 28, 29, 30, 31, 32, 33. Generally, these values near to zero represent the stability and fast convergence performance of the algorithm. This graph gives strong evidence that the proposed methodology all standard deviation results are near to zero which presents the proof of global optima solution stability and fast convergence performance of the proposed method outperform than others. Here, we can conclude that the proposed method is able to trap the best global optima with least number of iterations and time as comparison than others.

This paper has proposed a novel approach based on the recent meta-heuristic algorithm of SChoA. After using this approach in digital filter synthesis, it was found that, relative to other approaches, the proposed process has a greater ability to achieve optimal solutions in the same initial state (the initial population and the same number of iterations). In our suggested method, SChoA algorithm was applied separately to each node, where the location is modified according to the retiming strategy to which the SChoA algorithm is applied. In this way, the SChoA algorithm is applied to all the nodes available on the path. This enables the proposed algorithm to solve the current restrictions of identifying operator execution quickly and accurately. Lattice Ladder IIR filter system is taken into the account as an example to evaluate different outcomes using evolutionary algorithms based retiming approach. By significantly reducing the longest activities in a retimed DFG, the clock performance increases. It is by lowering while using this as next critical path in the system. The count of registers will rise in the process that could be the designer’s limit.

**Figure 20:**
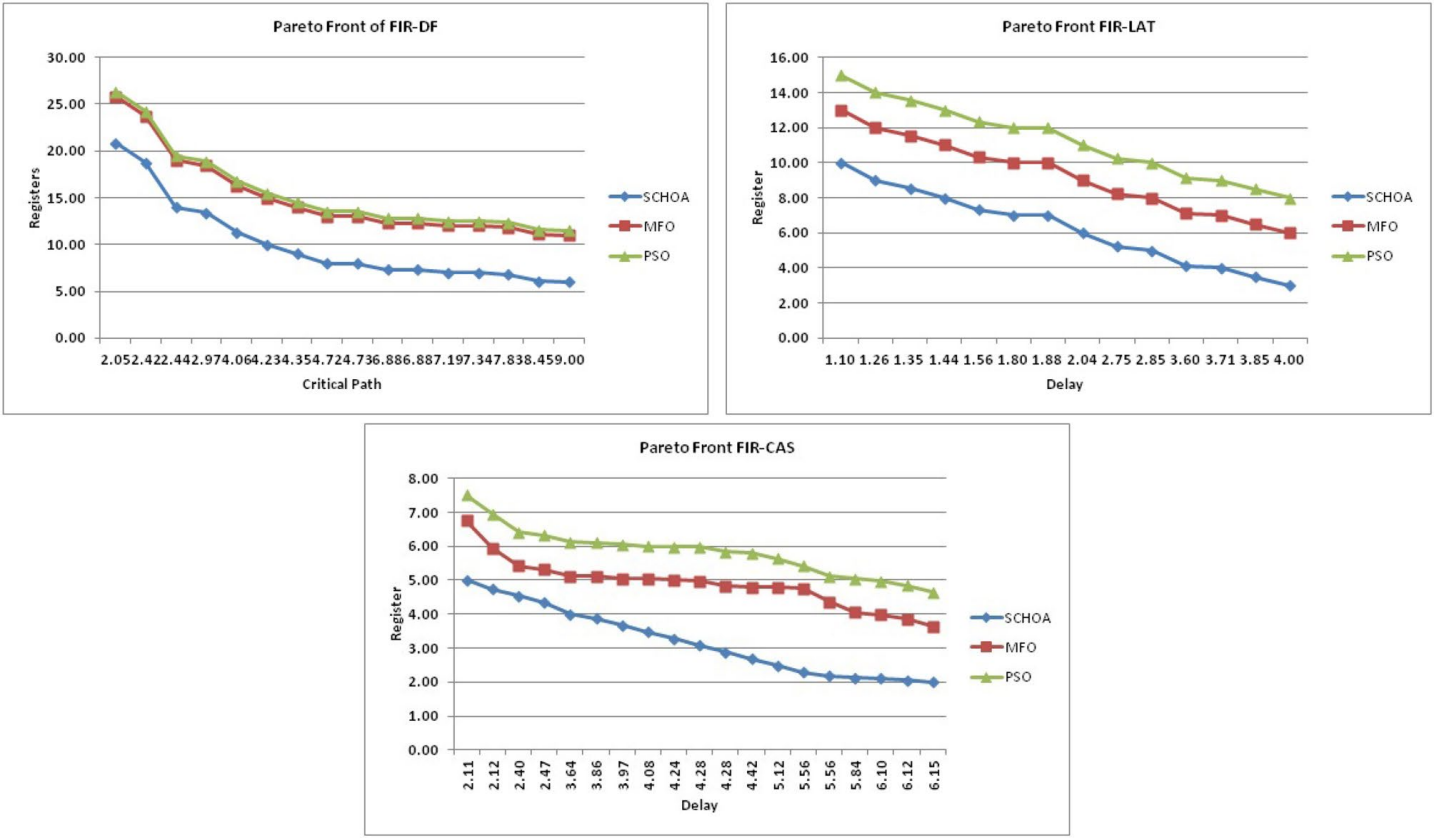
The pareto graph of algorithms on multi-objective DF-FIR, LATFIR and CASFIR digital filters.

**Figure 21:**
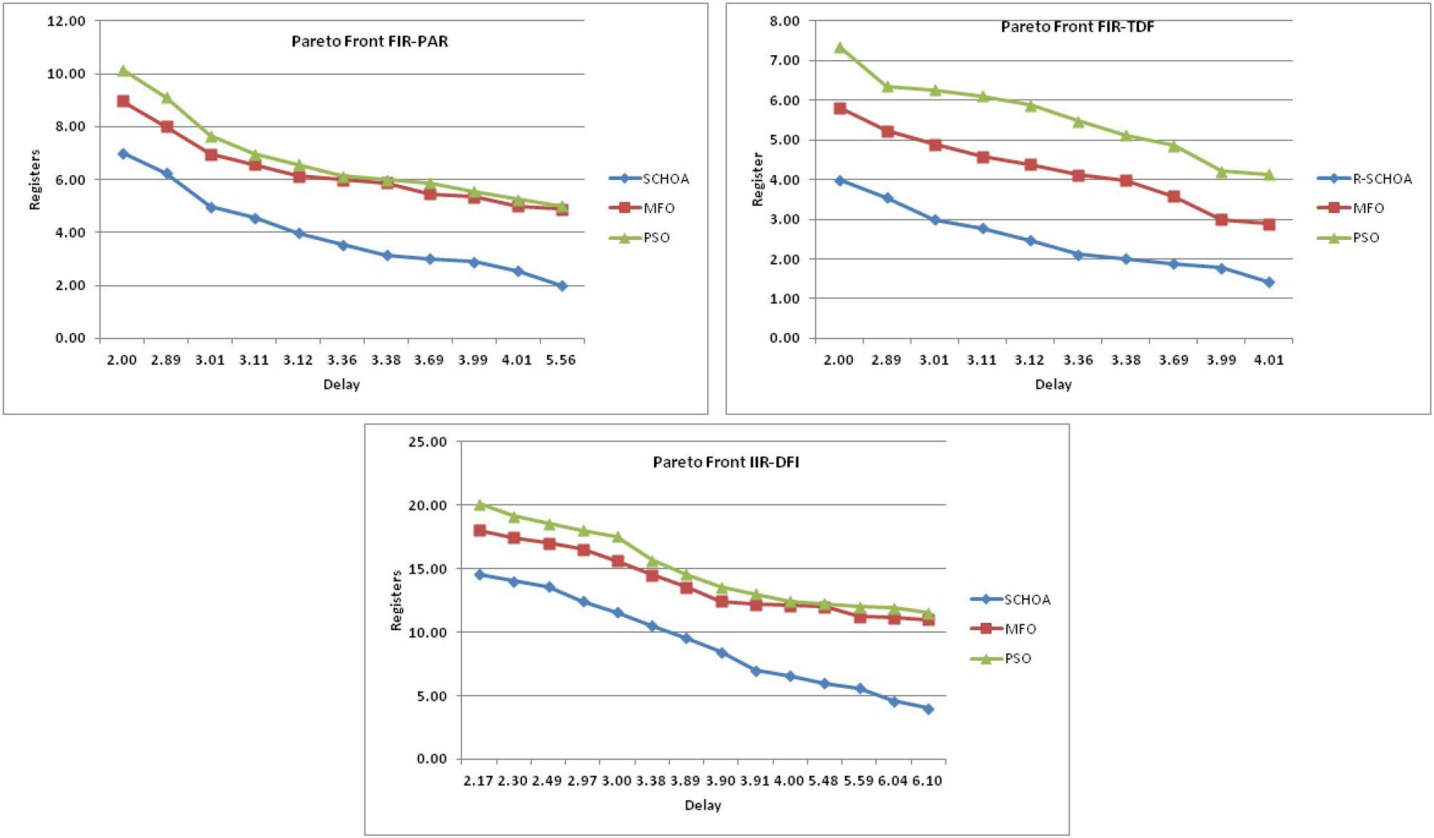
The pareto graph of algorithms on multi-objective PARFIR, TDFFIR and DF1IIR digital filters.

**Figure 22:**
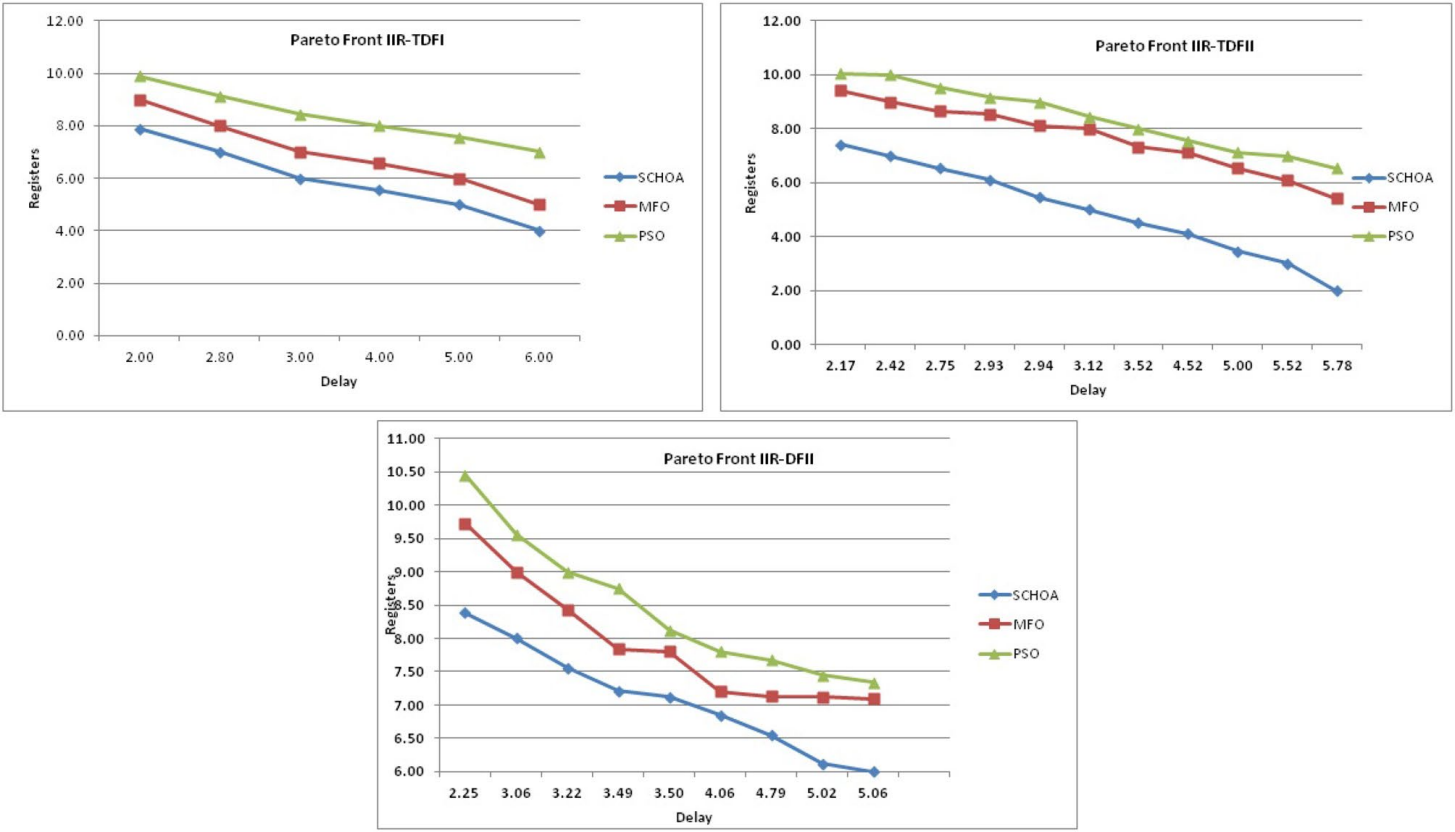
The pareto graph of algorithms on multi-objective TDF1IIR, TDF2IIR and DF2IIR digital filters.

**Figure 23:**
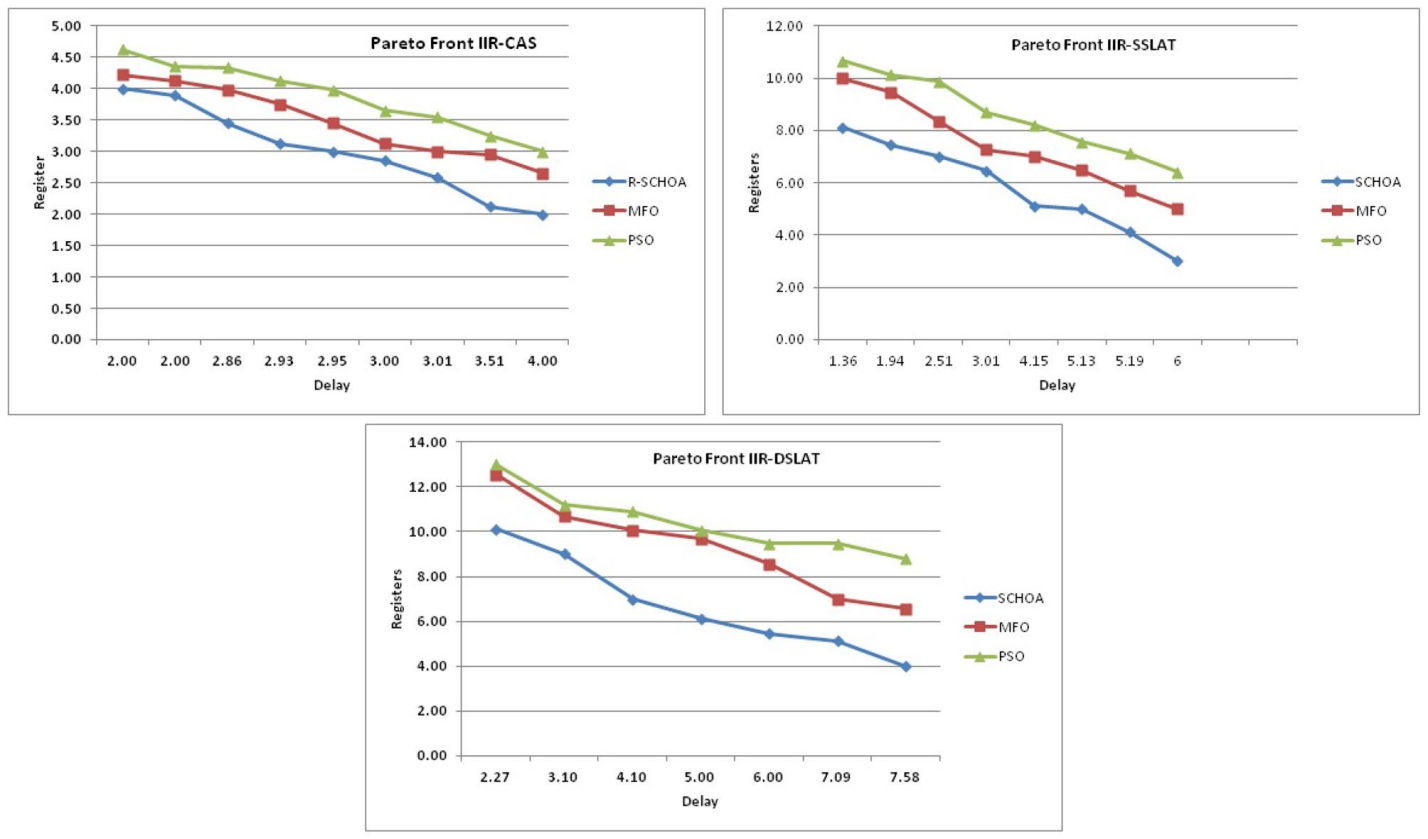
The pareto graph of algorithms on multi-objective CASIIR, SLATIIR and DLATIIR digital filters.

**Figure 24:**
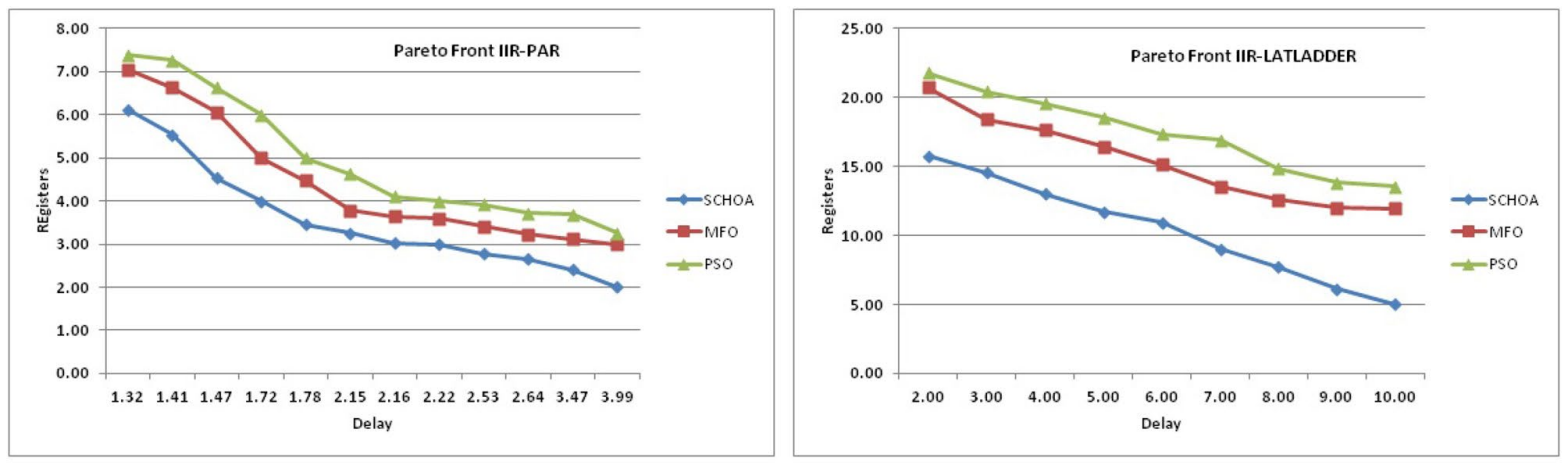
The pareto graph of algorithms on multi-objective PARIIR and LADIIR digital filters.

SChoA, MFO, PSO are highlighted here as the evolutionary algorithm based on Pareto, although there are other algorithms available on Pareto that may be considered for comparative analysis. The results of the algorithms have been illustrated in Table 35. In the entire solution space, the decision vectors that are not dominated can be represented as optimal Pareto and entail an optimal Pareto front. The Pareto front has been shown in two dimensions (Path delay and number of registers) to manipulate the objectives. The Pareto fronts identified using the information gathered for the analysed filters from the objective solution space are shown in the Figs. 20, 21, 22, 23 and 24 where the blue line indicates the relevant information obtained by the proposed process, while the red line reflects the algorithm data based on the MFO and the green line shows the algorithm-related data based on the PSO. In addition, the number of registers has been used in the vertical axis to clearly represent the data, which provides a clearer comparison of the three methods. In the Lattice Ladder IIR filter, the most appropriate solution fulfilling the goals is with a clock period of 5 time units and register count of 11. Besides that, even if clock period is chosen a limit, then with clock period as 3 and register count as 14 from the search space can be considered. From the design space if register count is a constraint, then response with clock time as 5 and register count as 11. An entry into the potential solution space would come from the one that does not alter the circuit functionality. This process will proceed until all feasible solutions are obtained. The designer may select either solution refers to a time units for critical paths and the registers count.

Consequently, the findings certainly showed selection in view of register and path latency, leading to an improvement in the design stage of the filters for the optimum solution. The solution space that provides the path lower than the initial critical path that fulfils all the retiming features is calculated by all feasible routes from the source node to the destination node. In the DLAT-IIR, the Pareto set at a path delay of 3 time units and a register count of 6 is the most suitable option that meets both objectives. However, if only a path delay is considered a restriction, the solution can be interpreted as a path delay with 3 and register 10 as a register count. For the register count as a restriction, another approach in terms of path delay as 5 and register count as 6. A 37.13%, 30.18% improvement in the MUF and area, 39.74% and 40.51% in relative to MFO, PSO based algorithms. The pareto optimal front for the consideration of 14-digital filters are shown in Figs. 20, 21, 22, 23 and 24. The retimed best optima results have been evaluated during this work for the single and multi-objective digital filters. Generally the search domain is finalized at each path from initial node to final node which provides the path less than the critical way that fulfills all the given conditions or properties.

**Table 35:** Pareto front result for retimed filters using different evolutionary algorithm for benchmark circuit.

Digital filters	SChoA		MFO		PSO	
F	FRC	FCP	FRC	FCP	FRC	FCP
DF-FIR	**10**	**2**	15	4	19	5
LATFIR	**6**	**2**	9	2	11	4
CASFIR	**3**	**3**	5	4	6	3
PARFIR	**4**	**3**	5	3	6	4
TDFFIR	**3**	**2**	3	3	4	3
DF1IIR	**8**	**3**	12	5	13	4
TDF1IIR	**7**	**4**	8	4	9	4
TDF2IIR	**4**	**3**	5	4	7	4
DF2IIR	**7**	**4**	8	4	8	4
CASIIR	**3**	**2**	4	3	4	3
SLATIIR	**5**	**2**	6	4	6	4
DLATIIR	**6**	**3**	10	5	10	5
PARIIR	**3**	**2**	4	2	5	3
LADIIR	**11**	**5**	15	5	18	5

[a] FRC: Feasible Register Count.

[b] FCP: Feasible Clock Period.

The bold values show the best solutions for problems.

**Table 36:** Comparison of SChoA- based method with MFO-based, PSO-based on different structures of digital filters.

Digital filters	SChoA		MFO^74^		PSO^75^	
F (-)	Max. freq (MHz)	No. of slices utilized	Max. freq (MHz)	No. of slices utilized	Max. freq (MHz)	No. ofslices utilized
DF-FIR	**160**.**78**	**27**	156.9	41	136.4	67
LATFIR	**180**.**48**	**54**	179.1	64	143.8	88
CASFIR	**117**.**183**	**124**	106.9	174	115.4	193
PARFIR	**115**.**76**	**84**	115.34	109	97.34	166
TDFFIR	**198**.**88**	**49**	177.54	51	165.48	64
DF1IIR	**73**.**2**	**63**	48.19	80	63.45	86
TDF1IIR	**84**.**116**	**48**	84.11	63	83.11	85
TDF2IIR	**75**.**48**	**28**	56.48	32	55.78	64
DF2IIR	**68**.**855**	**86**	68.85	102	68.85	102
CASIIR	**93**.**31**	**32**	83.31	46	80.31	46
SLATIIR	**93**.**375**	**64**	54.95	75	51.28	73
DLATIIR	**66**.**588**	**94**	38.56	156	36.15	156
PARIIR	**196**.**5**	**32**	194.56	49	188.45	63
LADIIR	**28**.**186**	**79**	20.96	109	16.58	193

The bold values show the best solutions for problems.

**Table 37:** Improvement of the SChoA-based method compared to MFO-based, PSO-based.

% Performance improvement				
Digital filters	MUF		Area	
F	MFO	PSO	MFO	PSO
DF-FIR	26.7	38.13	34.15	59.7
LATFIR	21.05	25.51	15.63	38.64
CASFIR	22.63	11.18	28.74	35.75
PARFIR	21.05	18.92	22.94	49.4
TDFFIR	12.02	20.18	3.92	20.31
DF1IIR	25.79	15.37	21.25	26.74
TDF1IIR	23.59	15.05	23.81	43.53
TDF2IIR	33.64	35.32	12.5	56.25
DF2IIR	24.71	61.29	15.69	15.69
CASIIR	38.54	54.72	30.43	30.43
SLATIIR	43.76	52.37	14.67	12.33
DLATIIR	37.13	30.18	39.74	40.51
PARIIR	14.75	10.11	34.69	49.21
LADIIR	34.48	43.95	27.52	59.07

The information obtained through the simulations of the three methods are listed in the tabulated form, i.e. SChoA-Proposed Method, MFO-based Method, PSO-based Method. In all the methods, the initial population and the maximum number of iterations are equal to 30 and 500. The results of the HLS of the digital filters have been tabulated in Table 36 where the maximum frequency available and the occupied area, that is the number of slices register used for the implementation of the operators and registers.

Table 37 summarises the percentage of the improvement achieved by the proposed SChoA-based method than other methods (MUF-based and PSO-based) while synthesising each digital filter. Table 37 clearly illustrates that in the DF-FIR, the current method have significantly improve MUF by 26.70% and 38.13% as compared to the MFO-based and PSO- based method. And the proposed method have provided improved MUF by 61.29% compared to the PSO-based method for DF2IIR. And compared to the MFO-based and PSO-based methods, the proposed method used fewer slices. For the optimum frequency, the proposed method in LAT FIR, DLATIIR filters synthesis revealed the best compared to the MFO-based, PSO-based, with 21.05%, 25.51% and 37.13%, 30.18% improvement. The best outcome for utilized slice registers was observed in the DF-FIR synthesis, with an improvement of 59.70% compared to the PSO-based method and an improvement of 39.74% in the DLAT-IIR synthesis compared to the MFO-based method. Important improvements have been identified in the Tables 36 and 37 to reach an optimal solution in terms of throughput. Our proposed method outperformed the other two with respect to two parameters (MUF and Area) for the filter synthesis. In Table 38, have been compared the execution time taken by the evolutionary algorithm for the 14- different digital filters. The results of this table give strong evidence that the proposed algorithm is able to tackle these issues in least time as comparison with others.

**Table 38:** Comparison of execution time for the various benchmark circuit.

Digital filters	SChoA	MFO	Reduction	PSO	Reduction
	Run time	Run time	$(\%)$	Run time	$(\%)$
DF-FIR	**1**.**57**	6.01	73	2.06	23
LATFIR	**0**.**768**	5.42	77	1.17	33
CASFIR	**0**.**913**	4.22	78	1.37	33
PARFIR	**0**.**993**	4.09	75	1.42	30
TDFFIR	**0**.**568**	2.71	75	0.92	36
DF1IIR	**1**.**03**	4.64	77	1.6	35
TDF1IIR	**1**.**02**	4.54	78	1.59	35
TDF2IIR	**1**.**06**	4.12	76	1.71	38
DF2IIR	**1**.**09**	4.36	79	1.59	45
CASIIR	**0**.**55**	2.73	78	0.91	39
SLATIIR	**0**.**68**	3.38	78	1.75	61
DLATIIR	**1**.**05**	4.98	78	1.73	39
PARIIR	**0**.**77**	3.5	78	1.19	35
LADIIR	**1**.**65**	6.3	76	2.17	23

The bold values show the best solutions for problems.

By comparing the performance of models, it would be reported that the optimum operating frequency for Lattice Ladder IIR filter has enhanced from 17.884 to 28.186 MHz that is improved by 57.6% whereas the number of slices used get declined by 23.52%. From the statistic it has been seen that the proportion of latches does get controlled well with desired clock period for the evolutionary retiming algorithm. For the performance analysis which including area and delay, models are evaluated and HLS has been used to optimize register transfer logic. The improvement in the clock rate of FIR and IIR digital filters during novel retiming algorithm are shown in Table 37. It highlights that after implementing the novel approach, the stepladder of the different arrangements are whittled down. The declination of the register count accelerates the design’s clock rate and trim down the feature size that further enhances performance level. Summing up, on the basis of all simulations, we concluded that the proposed methodology can tackle the complex digital filters issues strongly.

### HLS of digital filters design

Under this phase, the proposed strategy has been implemented on the high level of synthesis. HLS (high level synthesis) is a paramount phase during designing the digital filters. Normally, HL optimization decreases design period at minor stages, foremost to superior circuit indices^73^. HLS is a platform of very big scale integration (VLSI) design where in behavioral explanation is transformed into a physical representative^76,77^. The HLS is the initial stage in synthesizing a circuit and data flow graph (DFG) is utilized to illustrate the behavioral explanation, which defines the operators’ type and the connections amid them. The assumed DFG has been demonstrated by the Eq. (7.1);

7.1 $$\begin{aligned} Y=(((a+b) \times (c\times d))+((e+f)\times (g\times h)))+((e+f)\times (g\times h)) \end{aligned}$$

**Figure 25:**
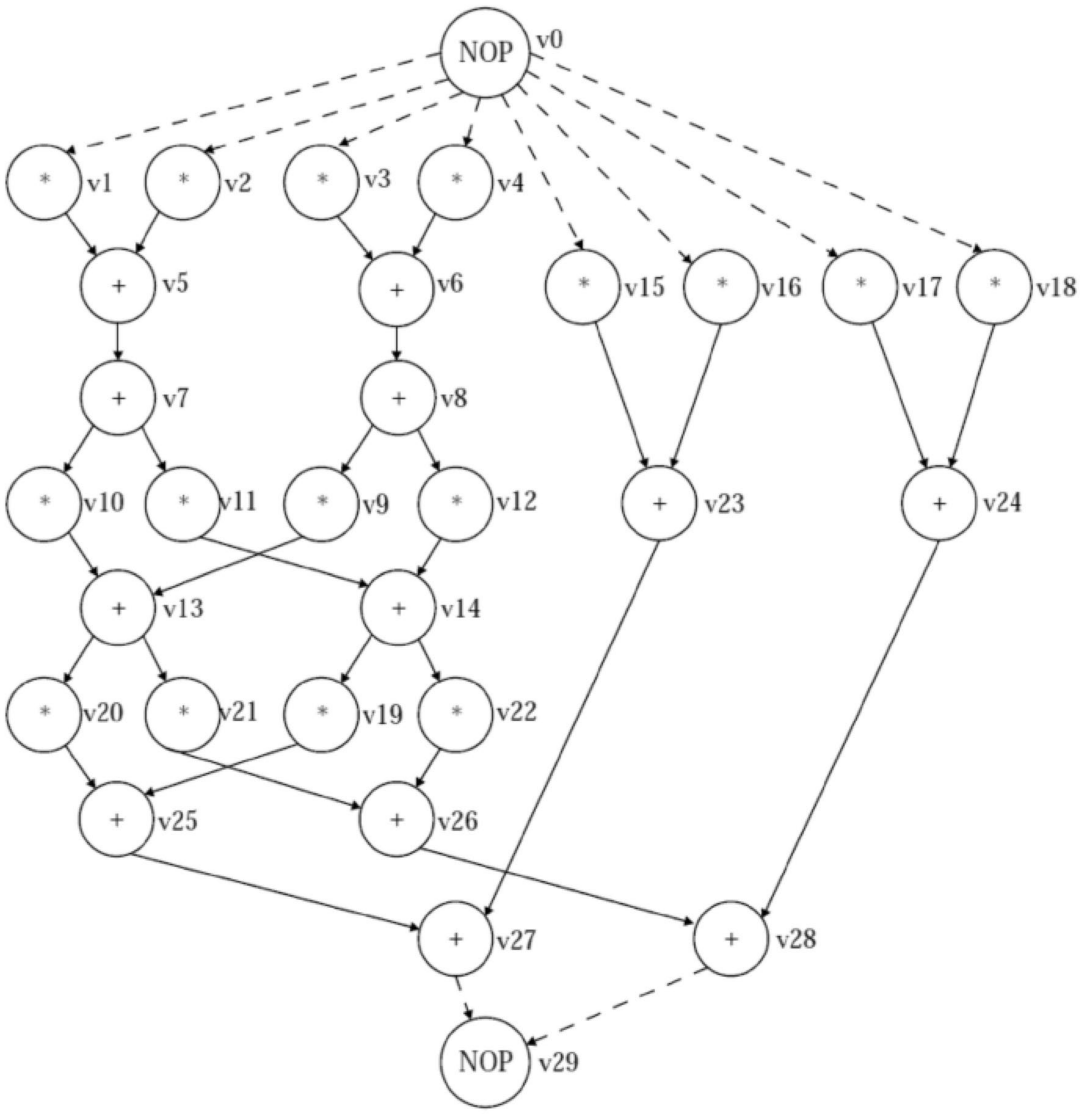
The ARF data flow graph.

The digital filters (DF’s) are commonly utilized for videos, process signals, images, communication applications, digital signal processing etc. The auto regressive filter (ARF), finite impulse response (FIR), the band-pass filter (BPF), the infinite impulse response (IIR), the elliptic wave filter (EWF) and the wave digital filter (WDF) are the DF’s used in this work. The DFG of the ARF used in this text has been demonstrated by Fig. 25^78^.

The following fitness function has been considered for evaluating the area, power and delay by the proposed strategy and verify the accuracy by the results of MFO^74^ and PSO^74^ algorithms:

7.2 $$\begin{aligned} F=w_{1} \times \frac{l_{t}}{l_{max}}+w_{2} \times \frac{a_{t}}{a_{max}}+w_{3} \times \frac{p_{t}}{p_{max}} \end{aligned}$$

where *F* is illustrated the fitness function, $w_{1}$,$w_{2}$,$w_{3}$ are describes the weights of the power, delay and area terms, $l_{t}$ is represented the schedule length of sample evaluated, $a_(t)$ is illustrated the total number of registers and transistors in the operators, $p_{t}$ is denoted the power consumption of operators, $l_{max}$ is denoted the long scheduled length in the current crowd,$a_{max}$ is denoted the largest area in the current crowd and $p_{max}$ is denoted the highest power in the current crowd respectively.

**Table 39:** Experimental outcomes of methods on IIR DFG filter.

Modes	Static	PSO			MFO			SChoA		
		Delay	Area	Power	Delay	Area	Power	Delay	Area	Power
$w_1=0.8$	Mean	5	6442.88	7007.66	5	5891.2	6349.97	**5**	**5489**.**3**	**6032**.**12**
$w_2=0.1$	sd	0	0.062	0.068	0	0.009	0.009	**0**	**0**.**006**	**0**.**007**
$$w_3=0.1$$										
$w_1=1$	Mean	7.42	3199.68	3217.85	7.08	3074.88	3142.2	**6.09**	**2711**.**43**	**3011**.**2**
$w_2=0.8$	sd	0.538	0.025	0.021	0.340	0.009	0.005	**0**.**263**	**0**.**005**	**0**.**003**
$$w_3=0.1$$										
$w_1=1$	Mean	7.3	3210.24	3187.59	7.14	3089.28	3142.2	**6**.**20**	**2756**.**09**	**3055**.**2**
$w_2=0.1$	sd	0.544	0.017	0.015	0.35	0.010	0.005	**0**.**28**	**0**.**008**	**0**.**003**
$$w_3=0.8$$

**Table 40:** Experimental outcomes of methods on FIR DFG filter.

Modes	Static	PSO			MFO			SChoA		
		Delay	Area	Power	Delay	Area	Power	Delay	Area	Power
$w_1=0.8$	Mean	9.3	7855.04	8084.97	9.08	7143.04	7324.19	**8**.**55**	**6345**.**09**	**7122**.**09**
$w_2=0.1$	sd	0.462	0.077	0.088	0.274	0.063	0.073	**0**.**211**	**0**.**039**	**0**.**049**
$$w_3=0.1$$										
$w_1=1$	Mean	15	3742.72	3222.89	14.84	3549.12	3157.33	**12**.**99**	**3410**.**67**	**3021**.**83**
$w_2=0.8$	sd	0.782	0.024	0.021	0.548	0.013	0.009	**0**.**4154**	**0**.**010**	**0**.**006**
$$w_3=0.1$$										
$w_1=1$	Mean	15.16	3723.2	3152.29	15.08	3694.08	3142.2	**12**.**08**	**3420**.**80**	**3027**.**2**
$w_2=0.1$	sd	0.618	0.001	0.008	0.274	0.01	0.005	**0**.**201**	**0**.**001**	**0**.**003**
$$w_3=0.8$$										

The bold values show the best solutions for problems.

**Table 41:** Experimental outcomes of methods on ARF DFG filter.

Modes	Static	PSO			MFO			SChoA		
		Delay	Area	Power	Delay	Area	Power	Delay	Area	Power
$w_1=0.8$	Mean	8.3	11,340.8	12,394.53	8.12	11,183.04	12,099.8	**7**.**17**	**10,387.90**	**11,890.2**
$w_2=0.1$	sd	0.505	0.023	0.027	0.328	0.004	0.004	**0**.**276**	**0**.**003**	**0**.**004**
$$w_3=0.1$$										
$w_1=1$	Mean	18.44	3627.2	3258.2	18.18	3543.04	3192.63	**17**.**16**	**3412**.**90**	**3032**.**29**
$w_2=0.8$	sd	0.787	0.016	0.017	0.388	0.011	0.014	**0**.**276**	**0**.**008**	**0**.**007**
$$w_3=0.1$$										
$w_1=1$	Mean	18.6	3688.32	3187.59	18.36	3575.04	3157.33	**17**.**38**	**3489**.**10**	**3064**.**66**
$w_2=0.1$	sd	0.670	0.014	0.014	0.485	0.12	0.009	**0**.**397**	**0**.**009**	**0**.**006**
$$w_3=0.8$$										

The bold values show the best solutions for problems.

**Table 42:** Experimental outcomes of methods on EWF DFG filter.

Modes	Static	PSO			MFO			SChoA		
		Delay	Area	Power	Delay	Area	Power	Delay	Area	Power
$w_1=0.8$	Mean	14	6924.8	6702.99	14	6902.4	6677.78	**14**	**6764**.**8**	**6489**.**65**
$w_2=0.1$	sd	0	0.006	0.007	0	0.007	0.008	**0**	**0**.**003**	**0**.**004**
$$w_3=0.1$$										
$w_1=1$	Mean	22.76	4039.04	3388.73	24.62	3873.6	3217.85	**23**.**16**	**3714**.**5**	**3176**.**02**
$w_2=0.8$	sd	5.057	0.043	0.057	4.347	0.016	0.021	**4**.**098**	**0**.**012**	**0**.**017**
$$w_3=0.1$$										
$w_1=1$	Mean	24.46	4070.08	3323.16	24.74	3954.56	3182.55	**23**.**43**	**3863**.**09**	**3049**.**12**
$w_2=0.1$	sd	3.95	0.043	0.057	3.433	0.15	0.013	**3**.**09**	**0**.**009**	**0**.**014**
$$w_3=0.8$$										

The bold values show the best solutions for problems.

**Table 43:** Experimental outcomes of methods on BPF DFG filter.

Modes	Static	PSO			MFO			SChoA		
		Delay	Area	Power	Delay	Area	Power	Delay	Area	Power
$w_1=0.8$	Mean	8.22	6996.48	7203.75	8.02	6458.88	6584.18	**6**.**57**	**6183**.**11**	**6209**.**32**
$w_2=0.1$	sd	0.545	0.062	0.069	0.141	0.021	0.022	**0**.**67**	**0**.**013**	**0**.**014**
$$w_3=0.1$$										
$w_1=1$	Mean	15.94	3884.48	3333.84	17.04	3628.8	3217.85	**16**.**57**	**3433**.**66**	**3100**.**2**
$w_2=0.8$	sd	3.449	0.033	0.027	4.035	0.02	0.016	**2**.**908**	**0**.**01**	**0**.**007**
$$w_3=0.1$$										
$w_1=1$	Mean	17.42	3870.72	3263.24	17.48	3728.96	3197.68	**16**.**06**	**3502**.**19**	**3001**.**20**
$w_2=0.1$	sd	3.038	0.025	0.025	2.894	0.28	0.014	**2**.**139**	**0**.**018**	**0**.**008**
$$w_3=0.8$$										

The bold values show the best solutions for problems.

**Table 44:** Experimental outcomes of methods on WDF DFG filter.

Modes	Static	PSO			MFO			SChoA		
		Delay	Area	Power	Delay	Area	Power	Delay	Area	Power
$w_1=0.8$	Mean	14.12	6904.64	6353.97	14.02	7040.96	6509.13	**13**.**08**	**6998**.**34**	**6423**.**09**
$w_2=0.1$	sd	0.385	0.063	0.082	0.141	0.028	0.037	**0**.**119**	**0**.**023**	**0**.**031**
$$w_3=0.1$$										
$w_1=1$	Mean	22.28	4310.08	3359.06	23.66	4184.32	3253.15	**22**.**67**	**4034**.**09**	**3110**.**45**
$w_2=0.8$	sd	5.789	0.019	0.032	4.926	0.016	0.024	**4**.**098**	**0**.**012**	**0**.**018**
$$w_3=0.1$$										
$w_1=1$	Mean	23.18	4342.72	3298.54	25.02	4257.28	3192.63	**24**.**75**	**4211**.**02**	**3009**.**29**
$w_2=0.1$	sd	5.283	0.016	0.029	4.321	0.012	0.014	**3**.**945**	**0**.**008**	**0**.**010**
$$w_3=0.8$$										

The bold values show the best solutions for problems.

**Figure 26:**
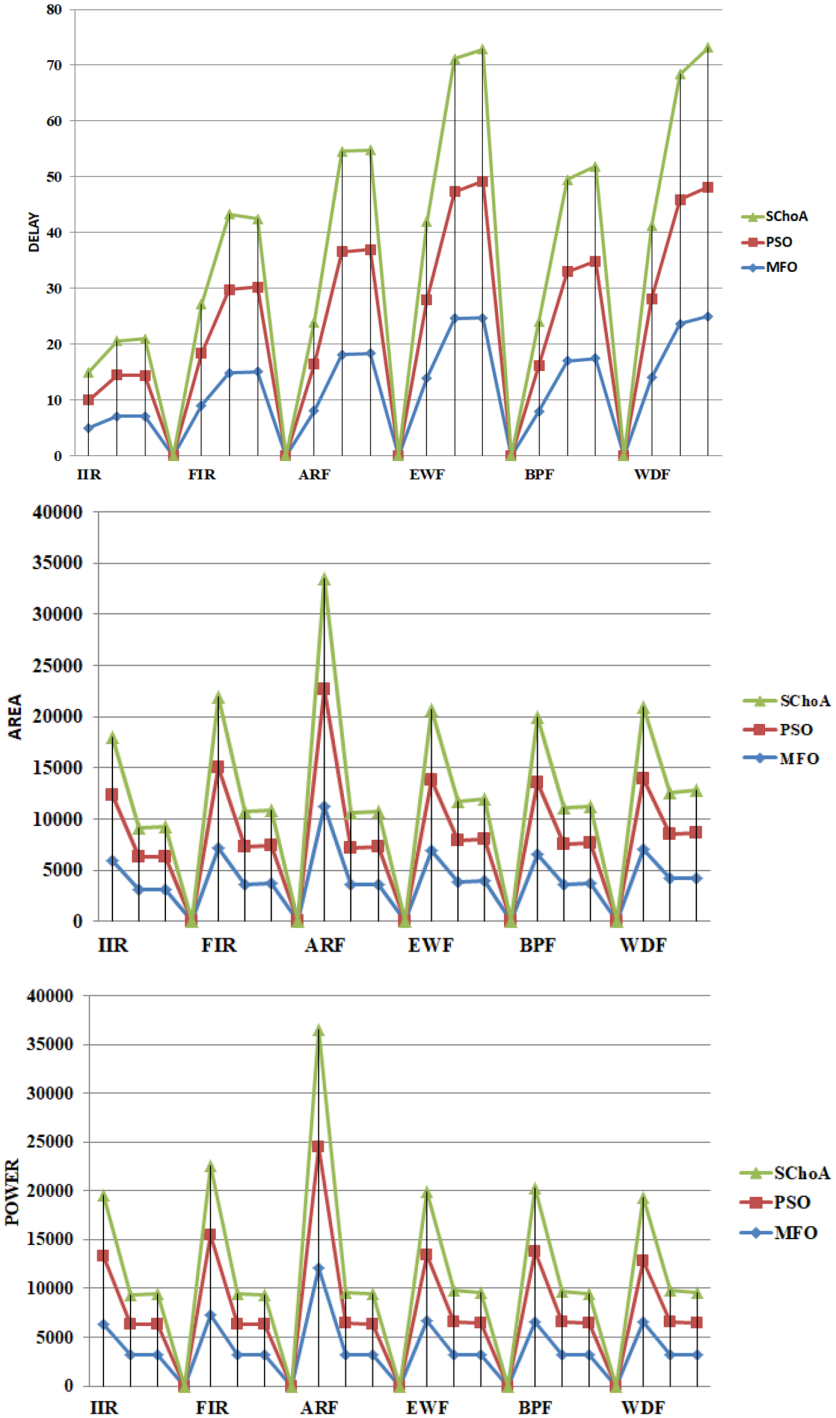
The best outcomes for delay, occupied area and power in HLS of digital filter issues.

Further accurately illuminates on the best optima results attained by the newly proposed method in the subsequent period of this subdivision. These outcomes have been confirmed over the recent literature solutions achieved by MFO^74^ and PSO^74^. The code of the algorithms have been runned on Matlab-R2015a under the system with 8GM of RAM and Intel (R) Core (TM) i3-8130 U processor. The various constants values have been fixed for getting the best outcomes viz total no ’s of search members are 30, total no’s of generations are 100, total no’s of operational units and sources are 5 etc. The experimental outcomes of the HLS of the digital filters have been described through Tables 39, 40, 41, 42, 43, 44. Similarly, the best outcomes of the newly developed approach for power, occupied area and lowest delay are presented by Fig. 26. All outcomes have assessed on three modes such as $w_1=0.8$, $w_2=1$,$w_3=1$ and $w_1=1$, $w_2=0.8$, $w_3=1$ and $w_1=1$, $w_2=1$, $w_3=0.8$ etc. For every mode, the average of the assimilated reaction for a 50-times effecting for the newly developed approach has been tabulated beside with their appropriate the standard deviation(*sd*). Here keep in mind that, the standard scores (*sd*) have been reported for a comparison and superior presentation of the result with respect to the power consumption and largeness of the occupied area.

The outcomes of Tables 39, 40, 41, 42, 43, 44, shows that the proposed method is able to give a highly accurate and superior outcome in terms of area, power and delay than others. All outcomes of IIR, FIR, ARF, EWF, BPF and WDF-DFG have been attained through changing the constant values of ($w_1$, $w_2$, $w_3$) and a major improvement in the global optimal outcome responses of the MA’s observed. For illustration, the best delay will be attained, linked to the other two modes, when supposing a weight of 0.8 for $w_{1}$, this factor is associated to the delay, and supposing a coefficient of 0.1 for $w_{2}$ and $w_{1}$ as the coefficients of the occupied area and power. The same is true for the other two modes. All least scores in Tables 39, 40, 41, 42, 43, 44 of the newly developed algorithm revealed are able to give the highly effective and accurate solutions for the occupied area and the least power consumption than MFO^74^ and PSO^74^ algorithms. In addition, the outcomes of Table 40 proved that the mean scores for the delay have been attained by the new method in comparison to others. These outcomes revealed that the new method is capable of decreasing the delay period than other for HLS issues. Therefore, the new strategy is competent to deliver the paramount outcome response in terms of delay, area and power consumption for HLS in VLSI circuits.

Summing up, the performance of the proposed algorithm shows that it is able to provide the high quality of the global optimal solutions outperforming the original algorithms. The powerful features of the proposed method can deal with the NP-hard applications of different domains. So this approach would be helpful in handling complex real-world problems.

## Conclusion and future work

In the paper, a enhanced version of chimp optimizer with sine cosine functions have been designed for the high level synthesis (HLS) of digital filter data-paths in terms of best score, execution time, occupied area and speed. The sine and cosine functions are helped of the algorithms in fluctuating toward or outward searching the global optima solutions. These functions are also able in ignoring the local optima and forcing for trapping the global optima fastly in the search domain. The performance of the algorithms have been tested on 23-standard test suites and 14-different digital filters of single and multi-objective functions in terms of minimum, maximum, average, standard deviation, execution time, occupied area and speed. The simulation results of the proposed strategy shows that the proposed strategy is able to successfully solve the high level synthesis of datapaths in digital filters problem in terms of area and speed respectively as comparison than to others. It is also able to trap highly accurate global optima solutions in the search area with least number of iterations and time than others.

Further, for the SChoA evolutionary algorithms, maximum improvement analysed in the frequency, the occupied area for DR- FIR is 38.13%, 59.70% and for DF2-IIR is 61.29%, 15.69% ,for LAT-IIR is 52.37% , 14.67% and also for LAD-IIR is 43.95%, 59.07%. This could greatly reduce the cost of systems with broad dimensions while increasing the design speed. Practically, the entire framework is saving the designer resources and time. In addition, the SChoA is competent to effectively solve the HLS of datapaths in digital filters issue in terms of lowest delay, area and power respectively than others. In future work, we shall develop the various enhanced versions of the algorithms for the high level synthesis and model identification of the digital filterings. In the end, we expect this work will encourage the young scientists of different domains, who are recently working on MA’s and digital filtering issues.

## Supplementary Information

Supplementary Information.

## Data Availability

All data included in this study are available upon request by contact with the corresponding author.
