# Supplementary material for: Enhanced chimp optimization algorithm for high level synthesis of digital filters
Source: Sci Rep. 2022 Dec 10;12:21389. doi: 10.1038/s41598-022-24343-x (PMC9741637; doi:10.1038/s41598-022-24343-x)
Supplement: Supplementary file 1 — Supplementary Information. [file 41598_2022_24343_MOESM1_ESM.pdf]

Appendix:

Table 1: 23-Standard Benchmarks test Functions

Key	Function formulation	$f(x^*)$	Category	Dimension	Range
f_1	$\sum_i^n x_i^2$	0	U	10,30,50,100	$x_i\varepsilon\,[-100,100]$
f_2	$\sum_{i=1}^n \lvert x_i\rvert + \prod_{i=1}^{x_i}\lvert x_i\rvert$	0	U	10,30,50,100	$x_i\varepsilon\,[-10,10]$
f_3	$\sum_{i=1}^n \left(\sum_{j=1}^i x_j\right)^2$	0	U	10,30,50,100	$x_i\varepsilon\,[-100,100]$
f_4	$max_i\left\{\lvert x_i, 1\leqslant i \le n\rvert\right\}$	0	U	10,30,50,100	$x_i\varepsilon\,[-100,100]$
f_5	$\sum_{i=1}^{n-1}\left[100\left(x_{i+1}-x_i^2\right)^2+(x_i-1)^2\right]$	0	U	10,30,50,100	$x_i\varepsilon\,[-30,30]$
f_6	$\sum_{i=1}^n (\lvert x_i+0.5\rvert)^2$	0	U	10,30,50,100	$x_i\varepsilon\,[-100,100]$
f_7	$\sum_{i=1}^n \left(ix_i^4+rand[0,1)\right)$	0	U	10,30,50,100	$x_i\varepsilon\,[-128,128]$
f_8	$\sum_{i=1}^n -x_i sin\left(\sqrt{\lvert x_i\rvert}\right)$	-418.9829×5	M	10,30,50,100	$x_i\varepsilon\,[-500,500]$
f_9	$\sum_{i=1}^n\left[x_i^2-10c0s\left(2\pi x_i\right)+10\right]$	0	M	10,30,50,100	$x_i\varepsilon\,[-5.12,5.12]$
f_{10}	$-20e^{\left(-0.2\sqrt{\frac{1}{n}\sum_{i=1}^n x_i^2}\right)}-e^{\left(\frac{1}{n}\sum_{i=1}^n cos(2\pi x_i)+20+e\right)}$	0	M	10,30,50,100	$x_i\varepsilon\,[-32,32]$
f_{11}	$\frac{1}{400}\sum_{i=1}^n x_i^2-\prod_{i=1}^n cos\left(\frac{x_i}{\sqrt{i}}+1\right)$	0	M	10,30,50,100	$x_i\varepsilon\,[-600,600]$
f_{12}	$\frac{\pi}{n}\left\{10sin\left(\pi y_i\right)+\prod_{i=1}^{n-1}\left(y_i-1\right)^2\left\lvert 1+10sin^2\left(\pi y_i+1+\left(y_{n-1}\right)^2\right)\right\rvert\right\}$	0	M	10,30,50,100	$x_i\varepsilon\,[-50,50]$
f_{13}	$0.1\left\{sin^2\left(3\pi x_i\right)+\sum_{i=1}^n\left(x_i-1\right)^2\left[1+sin^2\left(3\pi x_i+1\right)\right]+\left(x_n-1\right)^2\right.$	0	M	10,30,50,100	$x_i\varepsilon\,[-50,50]$
-	$\left.\left[1+sin^2\left(2\pi x_n\right)\right]\right\}+\sum_{i=1}^n\mu\left(x_i,5,100,4\right)$	-	-	-	-
f_{14}	$\left(\frac{1}{500}+\sum_{j=1}^{25}\frac{1}{\sum_{j=1}^2 (x_i-a_i)^6}\right)^{-1}$	1	F	2	$x_i\varepsilon\,[-65,65]$
f_{15}	$\sum_{i=1}^{11}\left[a_i-\frac{x_1\left(b_i^2+b_ix_2\right)}{b_i^2+b_ix_i+x_4}\right]^2$	0.0003	F	4	$x_i\varepsilon\,[-5,5]$
f_{16}	$4x_1^2-2.1x_1^4+\frac{1}{3}x_1^6+x_1x_2-4x_2^2+4x_2^4$	-1.0316	F	2	$x_i\varepsilon\,[-5,5]$
f_{17}	$\left(x_2-\frac{5.1}{4\pi^2}x_1^2+\frac{5}{\pi}x_1-6\right)^2+10\left(1-\frac{1}{8\pi}\right)cosx_1+10$	0.398	F	2	$x_i\varepsilon\,[-5,5]$
f_{18}	$\left[1+\left(x_1+x_2+1\right)^2\left(19-14x_1+3x_1^2-14x_2+6x_1x_2+3x_2^2\right)\right]$	3	F	2	$x_i\varepsilon\,[-2,2]$
-	$\times\left[\left(30+\left(2x_1-3x_2\right)^2\right)\left(18-32x_1+12x_1^2+48x_2-36x_1x_2+27x_2^2\right)\right]$	-	-	-	-
f_{19}	$\sum_{i=1}^4 c_ie^{-\sum_{j=1}^3 (x_j-p_{ij})^2}$	-3.86	F	3	$x_i\varepsilon\,[1,3]$
f_{20}	$\sum_{i=1}^4 c_ie^{-\sum_{j=1}^6 a_{ij}(x_j-p_{ij})^2}$	-3.32	F	6	$x_i\varepsilon\,[0,1]$
f_{21}	$-\sum_{i=1}^5\left[(X-a_i)(X-a_i)^T+c_i\right]^{-1}$	-10.1532	F	4	$x_i\varepsilon\,[0,10]$
f_{22}	$-\sum_{i=1}^7\left[(X-a_i)(X-a_i)^T+c_i\right]^{-1}$	-10.4028	F	4	$x_i\varepsilon\,[0,10]$
f_{23}	$-\sum_{i=1}^{10}\left[(X-a_i)(X-a_i)^T+c_i\right]^{-1}$	-10.5363	F	4	$x_i\varepsilon\,[0,10]$
